# Synthetic rhizosphere bacterial communities induce systemic resistance to barley powdery mildew without major shifts in the native bacterial community

**DOI:** 10.3389/fmicb.2026.1818676

**Published:** 2026-06-30

**Authors:** Linda Rigerte, Anna Sommer, A. Corina Vlot, Luis Daniel Prada-Salcedo, Thomas Reitz, Anna Heintz-Buschart, Mika T. Tarkka

**Affiliations:** 1 Department of Ecology of Agroecosystems, Helmholtz Centre for Environmental Research – UFZ, Halle (Saale), Germany; 2 Chair of Crop Plant Genetics, Faculty of Life Sciences: Food, Nutrition and Health, University of Bayreuth, Kulmbach, Germany; 3 Helmholtz Munich, Institute of Biochemical Plant Pathology, Neuherberg, Germany; 4 German Centre for Integrative Biodiversity Research (iDiv) Halle-Jena-Leipzig, Leipzig, Germany; 5 Department of Applied Microbial Ecology, Helmholtz Centre for Environmental Research – UFZ, Leipzig, Germany; 6 Crop Research Unit, Institute of Agricultural and Nutritional Sciences, Martin Luther University Halle-Wittenberg, Halle (Saale), Germany; 7 Biosystems Data Analysis, Swammerdam Institute for Life Sciences, University of Amsterdam, Amsterdam, Netherlands

**Keywords:** barley, *Blumeria graminis* f. sp. *hordei* (*Bgh*), PGPR-induced systemic resistance (ISR), powdery mildew, synthetic microbial communities

## Abstract

**Introduction:**

Synthetic microbial communities (SynComs) could help plants withstand biotic stress and reduce the need for pesticides. However, it remains unclear whether SynComs composed of host- or non-host-associated rhizosphere bacteria can trigger induced systemic resistance (ISR) in barley without causing major shifts in the native rhizosphere bacterial community.

**Methods:**

Here, we constructed two SynComs with known strain composition, composed of bacterial strains isolated from the host-associated barley rhizosphere and non-host-associated wheat rhizosphere. Their ability to trigger induced systemic resistance (ISR) against the barley powdery mildew pathogen Blumeria graminis f. sp. hordei (Bgh) was tested. To investigate plant-microbe interactions from both plant and microbial perspectives, we quantified Bgh propagation in leaves by DAF staining, analysed leaf transcriptomes, and profiled the rhizosphere microbiome using 16S rRNA gene amplicon sequencing and metatranscriptomics.

**Results:**

Both SynComs reduced fungal growth in barley leaves to a similar extent as the positive control strain, Pseudomonas simiae WCS417r, suggesting that ISR-like protection can also be achieved by defined multi-strain communities. Although both SynComs provided similar overall protection, the barley SynCom exhibited the strongest numerical reduction in fungal growth. These findings build on previous single-strain ISR studies and suggest that community-mediated protection is not restricted to host-derived bacterial consortia. Inoculations with both SynComs and WCS417r were not associated with statistically significant changes in the rhizosphere bacterial community structure. All treatments induced only subtle pre-infection transcriptional responses in barley leaves that were consistent with ISR-mediated priming. However, treatment with WCS417r yielded a higher number of differentially expressed genes than either SynCom. Rhizosphere metatranscriptomics revealed treatment-specific functional shifts. The two features K05516 and PF02868 were affected by all three treatments, implying the existence of shared changes related to stress adaptation and microbial activity. OTUs matching the inoculated SynCom members were still present in the rhizosphere at harvest, suggesting the persistence of at least some of the introduced communities.

**Conclusion:**

Together, these findings suggest that SynCom-based ISR is potentially a more ecologically relevant approach to microbiome-mediated disease protection in barley.

## 1 Introduction

Plant-associated microbial communities have a positive effect on plant fitness, development, and stress tolerance (Mendes et al., 2013; Panke-Buisse et al., 2015; Lau and Lennon, 2012). Plant beneficial bacteria have been isolated and characterized from root, leaf and stem surfaces, as well as from plant tissues (Glick, 2014). These bacteria have been shown to promote plant growth by facilitating nutrient acquisition, producing phytohormones like indole-3-acetic acid, ethylene, and jasmonic acid (Kejela, 2023) and by priming plant immunity and resistance against pathogens (Fujiwara et al., 2016; Israr, 2023). Generating host-associated microbial culture collections has enabled plant growth promoting (PGP) bacteria to be used as bioinoculants in the form of single strains or as consortia via the synthetic microbial community (SynCom) approach (Niu et al., 2017). SynComs are defined as microbial consortia comprising selected commensal strains with PGP functions and these communities can be used to reconstruct a host microbiome in a controlled environment and to study host-microbe and microbe-microbe interactions (Northen et al., 2024).

Plants possess a complex innate immune system to defend itself against pathogenic bacteria, fungi, protists, nematodes, viruses and insect herbivores (Agrios, 2009; Chisholm et al., 2006; Spoel and Dong, 2012; Leach et al., 2017; Shen et al., 2023). When attacked by a pathogen, plant local signaling activates defenses at the infection site, while systemic signaling informs unaffected tissues and triggers defense responses throughout the plant (Vlot et al., 2021). More precisely, plant cells use pattern recognition receptors to recognize the microbe or pathogen–associated molecular patterns. This process evokes pattern-triggered immunity, which together with effector-triggered immunity can induce salicylic acid signaling and activate systemic acquired resistance (SAR) (Vlot et al., 2021; Pieterse et al., 2012).

Besides SAR, which is triggered by pathogen attack, plants can develop induced systemic resistance (ISR), a beneficial bacteria-mediated immunity pathway first observed with *Pseudomonas* and *Bacillus* strains (Van Wees et al., 2008). ISR primes the plants into an “alerted” state, allowing faster and stronger responses to later pathogen attack (Heil, 2001; Conrath et al., 2015; Martinez-Medina et al., 2016). It is suggested that priming is effective against a wide range of pathogens, including (hemi-)biotrophic and necrotrophic types, and even herbivores (Vlot et al., 2021). Commensal *Pseudomonas* strains have been used to show ISR to depend on jasmonic acid and ethylene signaling pathways (Vlot et al., 2021; Pieterse et al., 2012; Yu et al., 2022), while pathways elicited by *Bacillus* and *Streptomyces* may also employ salicylic acid signaling (Kloepper et al., 2004; Ebrahimi-Zarandi et al., 2022; Van Wees et al., 2008; Kurth et al., 2014). ISR results in either subtle (Heil and Bostock, 2002) or extensive (Kurth et al., 2014) changes in gene expression in the unaffected tissues that prime the plant for an improved defense response upon attack by a pathogen. Although ISR is mainly elicited in the roots through interaction with beneficial rhizobacteria, it can protect above-ground tissues, such as leaves, by priming JA- and ethylene-dependent defense pathways (Pieterse et al., 1998; Van Wees et al., 2008). In barley, ISR and other beneficial effects have so far mainly been demonstrated for individual rhizobacterial strains, such as *Ensifer meliloti*, *Pantoea* sp., and *Pseudomonas* sp. However, more recent studies also suggest protective effects of less well characterized rhizobacteria, as well as induced responses that extend beyond defense to include nutritional pathways (Duan et al., 2023; Molitor et al., 2011; Mbaluto and Zytynska, 2025). Nevertheless, the capacity of defined multi-strain communities to trigger ISR in this host remains largely unexplored. Thus far ISR in barley has been largely studied using individual bacterial strains like *Pseudomonas simiae* WCS417r. Defined SynComs represent a conceptually distinct approach because they incorporate ecological and functional diversity, including potential microbe–microbe interactions, metabolic complementarity, and multiple ISR elicitors operating simultaneously. Unlike single-strain inoculants, SynComs may more accurately reflect natural rhizosphere processes and provide more robust or resilient microbiome-mediated protection in variable environmental conditions.

*Blumeria graminis* f. sp. *hordei* (*Bgh*) is a widespread fungal pathogen that causes the fungal disease powdery mildew. It affects a wide range of plants, including cereal crops like barley (Sacharow et al., 2023) and has also been reported in several barley growing regions (Wang et al., 2023; Cieplak et al., 2022; Cowger et al., 2018; Kloppe et al., 2022). *Bgh* affects plant leaves, and thus can have an adverse impact on crop yield (Czembor and Czembor, 2023) and photosynthesis (Coghlan and Walters, 1992), with warm and moist weather conditions favoring the pathogen (Elagamey et al., 2023; Bhardwaj et al., 2021). Rhizobacterium *Pseudomonas simiae* WCS417r has been shown to protect barley from *Bgh* and thus is commonly used for ISR studies (Sommer et al., 2026; Pieterse et al., 2021), to confer ISR in various host plants (Pieterse et al., 2021; Zhu et al., 2022).

Plants are strongly affected by climate change, including rising temperatures and prolonged droughts (Sato et al., 2024). Drought increases the susceptibility of plants to disease by reducing photosynthesis, transpiration and plant nutrient uptake (Hatfield and Prueger, 2015; Nievola et al., 2017). Such environmental changes may also facilitate the dispersal of plant diseases and pests (Delgado-Baquerizo et al., 2020), potentially resulting in increased crop damage and yield losses (Rezaei et al., 2023; Singh et al., 2023). These challenges highlight the need for sustainable strategies to enhance plant resilience to abiotic and biotic stresses, and ISR can serve as an important tool to maintain plant health, agricultural productivity, and environmental sustainability under future climatic conditions.

For example, Bziuk et al. (2022) previously investigated ISR triggered by native barley microbial communities. They demonstrated that a suspension of the barley rhizosphere microbiome can enhance the immune response and reduce the incidence of powdery mildew disease. Building on this, we tested whether SynComs from the rhizospheres of barley and wheat–composed of bacterial strains enriched under drought stress (each comprising Firmicutes, Proteobacteria, and Actinobacteria)–could trigger ISR in barley plants against the same pathogen. We hypothesized that due to the presence of Firmicutes and Actinobacteria (Kloepper et al., 2004; Ebrahimi-Zarandi et al., 2022) (i) both SynComs would elicit an ISR to the same extent as the ISR-eliciting bacterium *P. simiae* WCS417r, but in a jasmonic acid-, ethylene- and salicylic acid-dependent manner, (ii) given that barley was used as the host plant in our experiment, the barley SynCom would elicit a stronger response than the wheat SynCom due to the plant host preference of rhizosphere microbiomes, and (iii) at harvest time, only a subset of SynCom members would remain active and abundant in the rhizosphere due to differences in rhizosphere competence and persistence.

## 2 Materials and methods

### 2.1 Collection of barley and wheat rhizobacteria

Barley and wheat rhizobacteria were sampled from growing cereals at the Global Change Experimental Facility (GCEF) in Bad Lauchstädt (51° 23′ 30N, 11° 52′ 49E). GCEF is a field research station site of the Helmholtz Centre for Environmental Research^1^ that has a Haplic Chernozem soil type (Altermann et al., 2005). The site has a temperate continental climate with a mean annual temperature of 9.7 °C and mean annual precipitation of 438 mm (1993–2013) (Schädler et al., 2019). The bacterial strains used in this study were sampled from organic and conventional farming plots that were exposed to an ambient and future climate treatment. The latter comprises besides warming a 10% increase in precipitation in spring (March–May) and autumn (September–November), as well as a rainfall reduction by 20% in summer (June–August). This simulates the climate expected for Bad Lauchstädt in 2080 (Schädler et al., 2019). The strains were isolated from the rhizospheres of the barley cultivar “Antonella” and the wheat cultivar “RGT Reform” in two different stages of plant development, stem elongation stage BBCH 37–39 and grain filling stage, BBCH 75–77. The isolation process is described in Breitkreuz et al. (2020) and Rigerte et al. (2025). Briefly, the rhizosphere was collected by gently detaching the soil from the plant roots, after which the organic material was removed manually and by sieving (2 mm). The soil was then mixed and suspended in double-distilled water. The bacteria were separated from the soil by sonication, and a suspension was inoculated on Pikovskaya’s (1948) agar medium. Plates were then incubated for three weeks at 25 °C and colonies showing phosphate solubilization (observed as clear halos around them) were purified.

### 2.2 Selection of bacterial isolates

**FIGURE 1:**
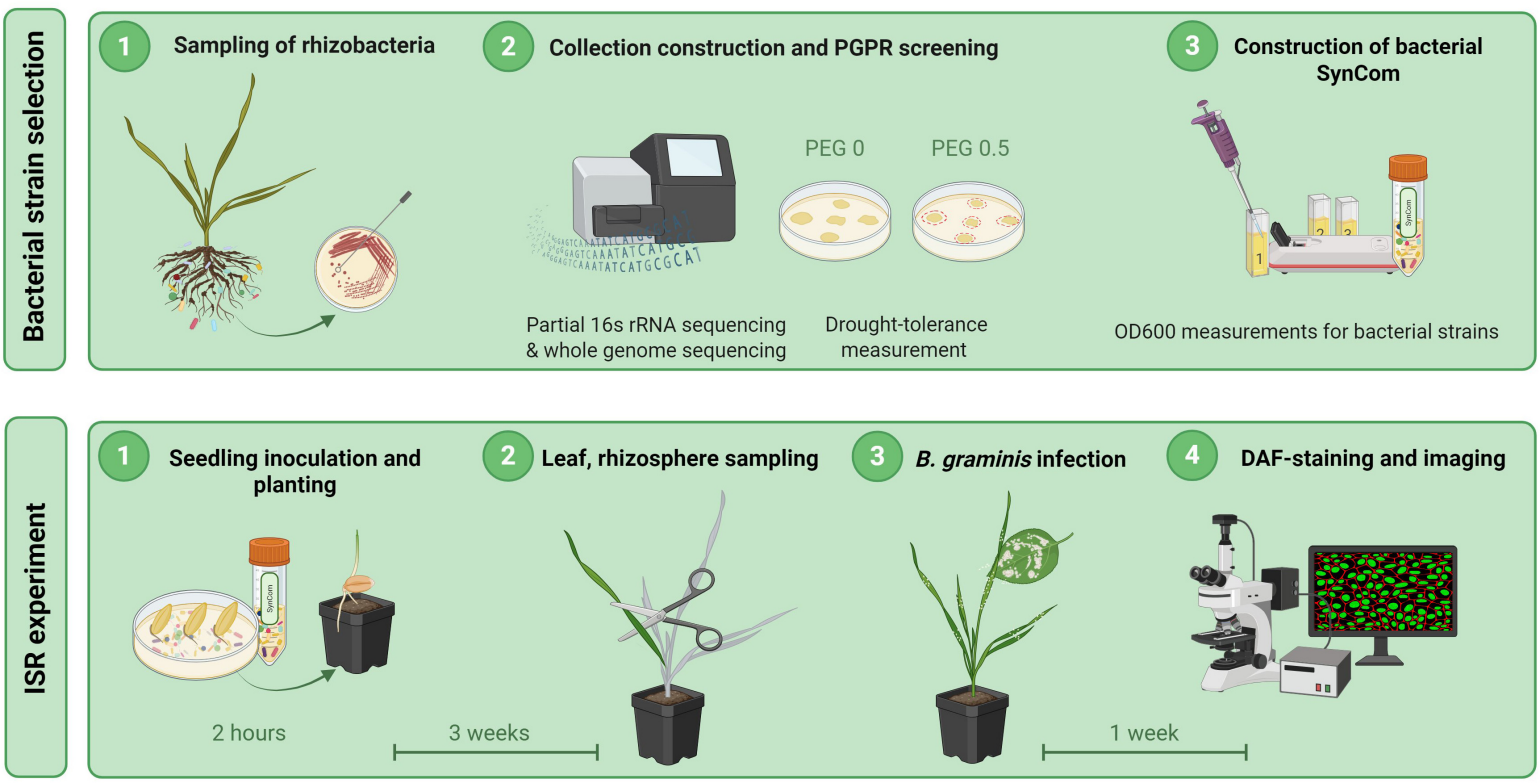
Workflow of the study, illustrating the work described in the “2 Materials and methods” sections “2.1 Collection of barley and wheat rhizobacteria,” “2.2 Selection of bacterial isolates,” and “2.5 Propagation of powdery mildew” [Created in BioRender. Rigerte (2026) https://BioRender.com/9kb2jl6].

Polyethylene glycol 8000 (PEG) was used to lower the water potential of agar medium and thereby assess the drought tolerance of the isolated strains. Drought stress was simulated by applying a 500 g/L solution of PEG (PEG0.5), which corresponds to an osmotic potential of approximately −1.1 MPa (Verslues et al., 2006). Drought tolerance was assessed by calculating the percentage reduction in colony diameter under PEG0.5 compared to PEG0. Genomic DNA was extracted by adding PEG to the bacterial suspensions and then vortexed with glass beads according to the method of Breitkreuz et al. (2020). DNA amplification was performed using PCR with the primers 27F and 1492R (Lane, 1991) and the Promega Green system (Promega, Madison, WI, USA). Subsequently, partial 16S rRNA gene sequences were obtained by Sanger sequencing using the primer BAC 341F at LGC Genomics (Berlin, Germany). The main objective of Sanger sequencing was to obtain sequence regions overlapping with ASVs obtained by 16S rRNA gene amplicon sequencing in previous studies. These amplicon sequencing datasets were generated from barley and wheat rhizosphere samples and were used as a reference for selecting bacterial strains for SynCom construction. Amplicon sequence variants (ASVs) that were more frequent under future climate conditions compared to ambient conditions in barley and wheat rhizosphere samples (Breitkreuz et al., 2021a), as well as ASVs enriched under drought compared to well-watered conditions in a greenhouse experiment using the same chernozem soil (Breitkreuz et al., 2021b), were selected from these datasets. These ASVs were then compared to the 16S rDNA sequences from our cultured bacterial collection to find isolates with identical or highly similar sequences. From this group, sixteen PEG-tolerant strains that showed similar growth on PEG0 and PEG0.5 were selected to assemble the Drought-Tolerant Synthetic Community (DT-SynCom). A full overview of the study workflow is presented in Figure 1.

### 2.3 Composition of a synthetic microbial community (SynCom) from barley and wheat

The strains were selected based on their drought tolerance and 16S rDNA sequence identity as described in sections “2.1 Collection of barley and wheat rhizobacteria” and “2.2 Selection of bacterial isolates.” The bacterial strains included in both SynComs were described in a previous study (Rigerte et al., 2025) and are listed in Table 1 to document the methods used in this work. Each SynCom was constructed by combining equal volumes of single-strain cultures as described in the following section.

**TABLE 1:** Composition of barley and wheat SynComs (refer to “2 Materials and methods” for details).

Strain ID	SynCom	Host origin	Genus	Species name*	Acc. number (NCBI)
B1	Barley	Barley	*Aeromicrobium*	*Aeromicrobium ginsengisoli*	NR_041384.1
B2	Barley	Barley	*Arthrobacter*	*Arthrobacter humicola*	NR_041546.1
B3	Barley	Barley	*Mesorhizobium*	*Mesorhizobium ciceri*	NR_113894.1
B4	Barley	Barley	*Mycolicibacterium*	*Mycolicibacterium montmartrense*	NR_151955.1
B5	Barley	Barley	*Phyllobacterium*	*Phyllobacterium brassicacearum*	NR_043190.1
B6	Barley	Barley	*Phyllobacterium*	*Phyllobacterium loti*	NR_133818.1
B7	Barley	Barley	*Priestia*	*Priestia aryabhattai B8W22*	NR_133818.1
B8	Barley	Barley	*Priestia*	*Priestia megaterium NBRC 15308 = ATCC 14581*	NR_112636.1
B9	Barley	Barley	*Pseudomonas*	*Pseudomonas poae*	NR_028986.1
B10	Barley	Barley	*Rhizobium*	*Rhizobium alamii*	NR_042687.1
B11	Barley	Barley	*Rhizobium*	*Rhizobium viscosum*	NR_042253.1
B12	Barley	Barley	*Rhodococcus*	*Rhodococcus fascians*	NR_037021.1
B13	Barley	Barley	*Streptomyces*	*Streptomyces nigrescens*	NR_117748.1
B14	Barley	Barley	*Streptomyces*	*Streptomyces resistomycificus*	NR_117748.1
B15	Barley	Barley	*Streptomyces*	*Streptomyces rishiriensis*	NR_112392.1
B16	Barley	Barley	*Streptomyces*	*Streptomyces tauricus*	NR_112405.1
W1	Wheat	Wheat	*Buttiauxella*	*Buttiauxella sp.*	MK638102
W2	Wheat	Wheat	*Pedobacter*	*Pedobacter* sp.	MK638129
W3	Wheat	Wheat	*Arthrobacter*	*Arthrobacter* sp.	MK638058
W4	Wheat	Wheat	*Bacillus*	*Bacillus* sp.	MK638067
W5	Wheat	Wheat	*Mesorhizobium*	*Mesorhizobium* sp.	MK638163
W6	Wheat	Wheat	*Chitinophaga*	*Chitinophaga* sp.	MK638107
W7	Wheat	Wheat	*Arthrobacter*	*Arthrobacter* sp.	MK638059
W8	Wheat	Wheat	*Pseudomonas*	*Pseudomonas* sp.	MK637939
W9	Wheat	Wheat	*Plantibacter*	*Plantibacter* sp.	MK638378
W10	Wheat	Wheat	*Pseudomonas*	*Pseudomonas* sp.	MK637993
W11	Wheat	Wheat	*Microbacterium*	*Microbacterium* sp.	MK638191
W12	Wheat	Wheat	*Inquilinus*	*Inquilinus* sp.	MK638140
W13	Wheat	Wheat	*Variovorax*	*Variovorax* sp.	MK638657
W14	Wheat	Wheat	*Streptomyces*	*Streptomyces* sp.	MK638538
W15	Wheat	Wheat	*Phyllobacterium*	*Phyllobacterium* sp.	MK638310
W16	Wheat	Wheat	*Ensifer*	*Ensifer* sp.	MK638128

*Species unresolved based on 16S rRNA gene sequence; designated as genus sp.

### 2.4 Preparation of the drought-tolerant synthetic community (DT-SynCom)

Bacterial strains from the rhizosphere of barley and wheat were cultivated for three days at 25 °C on yeast malt extract (YME) agar. The YME medium was prepared by dissolving the constituent ingredients (7 g malt extract, 2.8 g yeast extract, 10.5 g agar (2%) and 2.8 g glucose) in 700 ml water and autoclaving for 20 min. Each strain was resuspended in 10 mM magnesium chloride (MgCl_2_) until the optical density (OD) at 600 nm reached a value of 0.5. All individual suspensions were then centrifuged, the supernatant decanted and the bacterial pellets resuspended in 1 ml MgCl_2_. The barley strains were combined in equal proportions into a synthetic community and the wheat strains into a separate community, with each strain adjusted to a final concentration of 10^9^ cfu/mL in 10 mM MgCl_2_.

### 2.5 Propagation of powdery mildew

Barley seeds of the cultivar “Golden Promise,” which is susceptible to *Blumeria graminis* f. sp. *hordei*, were sterilized (as described in 3.6 excluding the surface sterilization step with 75% EtOH), sown and grown for three weeks under a 14/10-h day/night cycle at 20 °C/16 °C. Barley plants were then inoculated with *Bgh* as described below (Lenk et al., 2018, 2019). Plants were then grown for an additional ten days to obtain mature powdery mildew spores, which were used for infections. Propagation was repeated once a week to maintain a viable powdery mildew culture.

### 2.6 Inoculation of barley plants with SynComs and powdery mildew infection assay

Plant inoculation and powdery mildew infection assays were performed following established protocols (Sommer et al., 2024, 2026), with minor modifications as described below. Barley seeds were first surface sterilized by a one-minute wash with 75% ethanol (25 inversions per minute), followed by a five-minute incubation in sodium hypochlorite (NaClO). The washing step was followed by a ten-minute incubation in sterile water and repeated three times. The seeds were then placed on sterile NB plates (Supplementary Table 1) and kept in the dark at room temperature for germination. After four days, the seeds were divided into four groups and inoculated by means of a two hours long incubation with one of the following treatments: the wheat SynCom, the barley SynCom, or WCS417r (a widely used model strain, here used as a positive control) {all suspended in 10 mM MgCl2 to a final OD600 of 0.2 [equaling ∼2 * 10^8^ colony forming units (CFU)/mL]}. Germinated seeds were inoculated with either freshly prepared SynComs or previously prepared SynComs that were stored at −80 °C in 25% glycerol and washed three times with MgCl_2_ (10 mM) to remove glycerol prior to use. The fourth group was inoculated with MgCl_2_ (10 mM) to serve as a mock control solution. The barley plants were then grown in standard potting soil (Einheits Erde; Classic Profisubstrate, Germany) for 3 weeks under the conditions as described before. On day 21, the rhizosphere of each plant was collected by carefully removing the root-attached soil by hand, and the second and third leaves of three separate plants were sampled and stored frozen until subsequent nucleic acid extractions. The remaining barley plants and a glass slide were then placed in a cardboard box and infected with *Bgh* by gently shaking the infected plant over the box, all treatments receiving identical spore-dispersal handling. To spread the spores, a styrofoam board was fanned ten times in all directions. After one hour, the spores deposited on the glass slide were counted to verify the spore density of approximately 30 spores per mm^2^. Plants were then grown for one week as described before to allow fungal structures to develop, after which leaves were harvested and infection intensity was evaluated by fluorescence-based quantification of fungal structures following DAF staining [see Section “2.7 Plant diaminofluorescein (DAF) staining”].

### 2.7 Plant diaminofluorescein (DAF) staining

Plant diaminofluorescein (DAF) staining was performed to visualize fungal hyphae in plant tissues, following established protocols (Lenk et al., 2018, 2019). The MES-KOH (morpholinoethanesulfonic acid-potassium hydroxide) 0.5 M stock solution was prepared by adding 21.32 g MES to 200 ml H_2_O and adjusting the pH to 5.7 with KOH. As MES is light-sensitive, the bottle was wrapped in aluminum foil and placed in the refrigerator until further use (the shelf life of the solution is one month). The prepared MES stock solution was then used to make the DAF-FM-DA working solution described in Supplementary Table 2. For staining, the second and third leaves of barley were selected, and 16 leaf disks per treatment of inoculated plants were immersed in 4 ml of working solution (without added DAF) for 45 min (in the dark), then the solution was removed and 4 ml of DAF solution was added. The leaf disks were then incubated in the dark for 45 min and then vacuum infiltrated 3 to 5 times to aspirate the solution into the plant tissue, followed by incubation under light for 105 min. The solution was then removed and 1 ml of working solution was added for a wash step to remove any remaining DAF-FM DA. The leaf disks were then dried on a paper towel and placed upside down on a 96-multiwell plate filled with 1% phytoagar (any meniscus formed by the agar medium was removed by scraping to obtain a uniform surface for fluorescence measurement). The plate was then sealed with qPCR-foil and the Keyence BZ-X800 fluorescence microscope was used to image the leaf disks. Fluorescence was measured using a BZ-X Filter GFP (model OP-87763) with an excitation filter of 470/40 nm, an emission filter of 525/50 nm and a dichroic mirror with a cutoff wavelength of 495 nm. Fluorescence was normalized for each replicate as the ratio of the measured intensity to the mean intensity of the empty controls. A small pseudocount was added for log10 transformation to avoid undefined values when intensity ratios were zero (pseudocount = 0.1 × smallest non-zero ratio observed). Values were then plotted as log10(ratio + pseudocount). Statistical significance was assessed using ANOVA and Tukey HSD tests on three biological replicates, each comprising 16 leaf disks per treatment.

### 2.8 Nucleic acid extraction and sequencing

RNA extraction: total RNA from barley rhizosphere samples was extracted using the RNeasy PowerSoil Total RNA Kit (Qiagen, Hilden, Germany) following the extraction protocol provided by the manufacturer. Sequencing was performed by Azenta Life Sciences (Leipzig, Germany) on an Illumina instrument after rRNA depletion in a paired-end configuration generating approximately 20 million paired-end reads of 150 bp read length per sample.

Host leaf gene expression: Second and third leaves of barley plant were sampled and ground using liquid nitrogen. Leaf RNA was extracted using the NucleoSpin RNA Plant Kit (Macherey-Nagel, Düren, Germany) and Agilent Bioanalyzer was used to determine the RNA integrity number (RIN) which was ≥ 7 for all samples. Samples were sequenced by Novogene (Düsseldorf, Germany) and Illumina sequencing was performed using a NovaSeq X Plus Series sequencing platform, which generated approximately 20 million paired-end reads of 150 bp length per sample (40 million reads in total).

Rhizosphere 16S rRNA gene amplicon sequencing: barley rhizosphere DNA was extracted using the DNeasy PowerSoil Pro Kit (Qiagen, Hilden, Germany). The V4 region of the 16S rRNA gene was amplified using the primers 515F (GTGYCAGCMGCCGCGGTAA) and 806R (GGACTACNVGGGTWTCTAAT) (Caporaso et al., 2011), each with Illumina overhang adapters (P5: TCGTCGGCAGCGT CAGATGTGTATAAGAGACAG; P7: GTCTCGTGGGCTCG GAGATGTGTATAAGAGACAG). The Illumina MiSeq platform with Reagent Kit v3 was used to generate an average of ∼200,000 read pairs per sample (2 × 300 bp).

All sequencing data generated in this study, including rhizosphere metatranscriptomes, rhizosphere 16S rRNA gene amplicon sequencing data, and host leaf RNA sequencing data, have been deposited in NCBI under BioProject ID PRJNA1405459.

### 2.9 Rhizosphere 16S rDNA amplicon sequencing bioinformatics and microbiome analysis

The 2 × 300 bp paired-end reads were processed using the Dadasnake pipeline v0.11 (Weißbecker et al., 2020; Callahan et al., 2016) with default parameters. The process included quality filtering and truncation of forward and reverse reads to 170 bp and 130 bp, respectively. Reads with a minimum quality score of 13 were retained and a maximum expected error rate of 0.2 for both read directions was allowed. Error models were learned and sequencing errors corrected using DADA2, which was followed by dereplication, which collapsed identical reads into amplicon sequence variants (ASVs). ASVs were subsequently clustered at 97% sequence identity to generate operational taxonomic units (OTUs) (Schloss, 2021; Estensmo et al., 2021). Chimeras were removed using DADA2’s consensus algorithm. Taxonomic classification of the produced OTUs was performed against the SILVA v138.1 database (Quast et al., 2013). Additionally, OTUs potentially associated with the SynCom strains were identified using MMseqs2’s easy-search module by requiring both a minimum sequence identity of 99% and a minimum sequence coverage of 99% between the OTU sequence and the corresponding 16S rDNA sequence from the SynCom genomes published by our group (Rigerte et al., 2025). Prior to downstream analysis, non-target and low-confidence taxa were removed in R v4.4.1 to focus the dataset on bacterial rhizosphere community members. These included plant-derived sequences (chloroplasts and mitochondria), non-bacterial taxa (fungi and algae), taxa not considered typical plant or soil associated rhizosphere bacteria in this context (Rickettsiales, and Piscirickettsiales), *Escherichia* spp., and taxonomic assignments explicitly marked as “unknown” and/or “unclassified.”

Further processing was performed using functions from the phyloseq v1.50 (McMurdie and Holmes, 2013), microViz v0.12.7 (Barnett et al., 2021), and vegan v2.7-1 (Oksanen et al., 2025) R packages. The OTU table was pre-processed using the tax_fix() function from microViz to fill in missing taxonomic ranks at higher levels from lower levels. OTUs with zero reads across all samples were removed. Alpha diversity metrics (Shannon, Chao1 and observed) were calculated using the estimate_richness() function from phyloseq without additional filtering of the OTU table. To calculate beta diversity, the data were first filtered to remove OTUs with fewer than five reads across all samples, with no phyla being removed. Samples with zero reads across all OTUs were then filtered out. The data were then rarefied to even depth using phyloseq’s rarefy_even_depth() function. Beta diversity was assessed using Bray–Curtis distances calculated upon these data as inputs. A Principal Coordinates Analysis (PCoA) biplot was created using data from phyloseq’s ordinate() function to examine the distribution of the samples along the first two principal coordinates. Beta dispersions of the samples were calculated using betadisper() from vegan and statistically significant pairwise differences in these were assessed using TukeyHSD() (Tukey’s Honestly Significant Differences) from the stats package. Pairwise Permutational Multivariate Analysis of Variance (PERMANOVA) between all treatments was conducted using the pairwise.adonis() function from the pairwiseAdonis v0.4.1 package (Arbizu, 2020), with Bray–Curtis distances as the input variable, 9999 permutations, and Benjamini–Hochberg (BH) multiple testing *p*-value correction. Alpha and beta diversity of the soil microbiome samples were analyzed with 1,390 and 1,246 operational taxonomic units (OTUs) across 20 samples, respectively.

### 2.10 Leaf transcriptome analysis

Sequenced reads were trimmed using Trimmomatic (Bolger et al., 2014) with the default parameters, and mapped to the barley reference genome *Hordeum vulgare* cv. Morex genome assembly v3 using HISAT2 (Kim et al., 2015). FeatureCounts (Liao et al., 2014) was used to assess the number of reads per gene.

Differential gene expression analysis was performed in R using the following pipeline. Gene counts obtained by featureCounts were used for differential expression analysis using the DESeq2 package (Love et al., 2014) and a DESeqDataSet object was constructed using the metadata table containing treatment information. Differential gene expression between different treatments was determined by fitting a negative binomial generalized linear model and performing Wald tests for the pairwise comparisons. The obtained *p*-values were adjusted using the Benjamini–Hochberg procedure and genes with *p*-value < 0.05 and an absolute log_2_ fold change ≥ 1 were considered to be significantly differentially expressed. Initially, a set of the 200 genes showing the strongest variation associated with the treatment was determined by using a variance-partitioning analysis. The z-scores for all treatments were then compared, and a heatmap showing the 50 genes with the strongest differences was created using the ComplexHeatmap package (Gu et al., 2017).

### 2.11 Rhizosphere metatranscriptome analysis

The raw metatranscriptomic reads were trimmed using Trimmomatic in paired-end mode and then processed in sequential mode with SqueezeMeta v1.6.2 (Tamames and Puente-Sánchez, 2019). Contigs were assembled *de novo* using MEGAHIT (k-mer sizes 41, 61, 81, 99, 121), and short contigs (< 200 bp) were filtered using prinseq. Taxonomic and functional annotations (minimum 50% identity) involved ORF prediction using Prodigal, rRNA/tRNA detection using Barrnap and Aragorn, and DIAMOND similarity searches against GenBank, eggNOG, and KEGG, supplemented by HMMER3 for Pfam. Read mapping for quantification was performed using Bowtie2, with default parameters.

The SQM files generated by the pipeline were imported into R and processed using SQMtools (Puente-Sánchez et al., 2020) and allied packages. These files contained read counts attributed to taxonomic annotations as well as functional annotations (i.e., features) against the KEGG, COG, and Pfam databases. Prior to analysis, raw counts (abundances) were converted to per-sample relative abundances (with non-finite values being set to zero) and filtered to retain features with at least 1 × 10^6^ relative abundance in at least 70% of the samples. Top ten functional features for the KEGG, COG, and Pfam databases grouped by treatment were visualized using the plotFunctions() function from SQMtools using TPM (transcripts per million) values as input. Phylum-level taxonomy summaries grouped by treatment were generated using plotTaxonomy() from SQMtools with the TPM values also. Bray-Curtis distances computed from the relative abundance values calculated earlier were then used to assess beta diversity and dispersion which were then visualized using a PCoA [using pcoa() from ape (Paradis et al., 2004)] and tested for statistical significance using TukeyHSD() as described earlier. A whole model PERMANOVA as well as pairwise PERMANOVAs were also conducted as described on the Bray–Curtis distances. Finally, differential gene expression analysis was performed using DESeq2 with default parameters (Love et al., 2014).

## 3 Results

To facilitate the interpretation of the experimental design, it is important to distinguish between the post-infection disease assay and the molecular profiling datasets. Section “3.1 Barley and wheat SynComs reduce propagation of *Blumeria graminis* f. sp. *hordei* (*Bgh*) in barley leaves” reports on the impact of root inoculation treatments on the propagation of *Blumeria graminis* f. sp. *hordei* (*Bgh*) in barley leaves following pathogen challenge. The transcriptomic, 16S rRNA gene amplicon, and rhizosphere metatranscriptomic analyses presented in Sections “3.2 SynCom inoculation does not significantly alter rhizosphere bacterial community structure,” “3.3 Limited treatment effects on barley leaf transcriptomes after batch correction,” “3.4 Bioinoculant treatments only induce subtle transcriptional responses in barley leaves,” “3.5 Rhizosphere metatranscriptomes show similar taxonomic and functional profiles across treatments,” and “3.6 Bioinoculants induce selective functional shifts in rhizosphere metatranscriptomes,” meanwhile, were based on samples collected prior to the *Bgh* infection assay. These analyses were used to evaluate how plants and the rhizosphere responded to treatments during the priming process and before interacting with the pathogen.

### 3.1 Barley and wheat SynComs reduce propagation of *Blumeria graminis* f. sp. *hordei* (*Bgh*) in barley leaves

**FIGURE 2:**
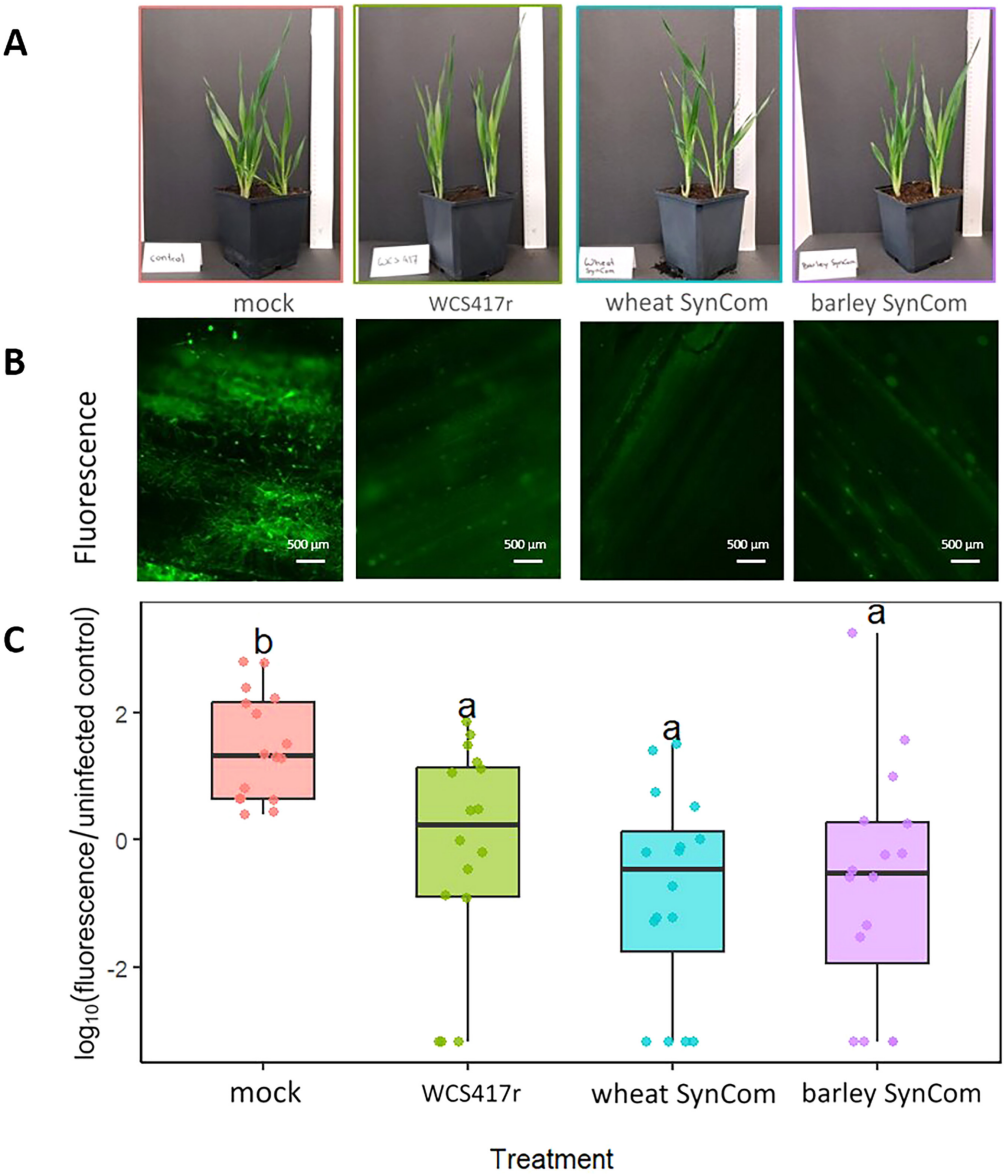
Barley and wheat SynComs enhance resistance against barley powdery mildew (*Bgh*). **(A)** Barley plants prior to *Bgh* infection, inoculated with either mock, WCS417r, wheat SynCom or barley SynCom inocula. **(B)** Representative fluorescence microscopy images of *Bgh* hyphae on leaf disks stained with DAF-FM-DA at 7 days post inoculation (dpi) in seedlings treated with mock, WCS417r, wheat SynCom and barley SynCom. **(C)** Quantification of *Bgh* on DAF-FM-DA-stained leaf disks, three biological replicates, each comprising 16 leaf disks per treatment. Fluorescence values were normalized to the mean intensity of empty wells. Values are shown as log10(ratio + pseudocount), where the ratio represents the normalized fluorescence intensity. Different letters indicate significant differences (*p* < 0.05) according to ANOVA followed by Tukey’s HSD test.

To assess whether the SynComs induce systemic resistance in barley, the roots of 21-day-old seedlings were inoculated with barley or wheat rhizosphere SynComs, or WCS417r, or a mock control, followed by *Bgh* inoculation. No clear differences in plant phenotype were observed among the treatments (Figure 2A). At 7 days post inoculation (dpi), plants treated with the three inoculants exhibited significantly lower fluorescence intensities compared to the mock-treated control group, indicating reduced *Bgh* growth (Figures 2B,C). Mean fluorescence was reduced by 50.4% in the barley SynCom treatment, 43.3% in the wheat SynCom treatment, and 34.9% in the WCS417r treatment, relative to the mock treatment. These results demonstrate that both the barley and wheat SynComs induce ISR in a manner similar to that of the ISR-eliciting bacterium WCS417r.

### 3.2 SynCom inoculation does not significantly alter rhizosphere bacterial community structure

Because the SynCom treatments reduced *Bgh* propagation in leaves, we next asked whether these ISR-associated treatments were accompanied by shifts in rhizosphere bacterial community composition, thus partial 16S rRNA gene amplicon sequencing was performed on rhizosphere samples collected at harvest. The rhizosphere microbiome comprised 1390 ASVs, dominated by the phyla Proteobacteria, Actinobacteriota, Bacteroidota, Firmicutes, Planctomycetota, Chloroflexi, Verrucomicrobiota, Myxococcota and Acidobacteriota, respectively (Supplementary Figure 1A). No statistically significant differences in either alpha or beta diversity were detected between treatments and the control (Supplementary Figures 1B,D), as supported by the PCoA ordination plot (Supplementary Figure 1C). Pairwise PERMANOVA comparisons showed small treatment effect sizes (*R*^2^ = 0.03–0.08) (Supplementary Table 3). OTUs with ≥ 99% sequence identity to the 16S rRNA gene sequences of the inoculated SynCom strains were detected in rhizosphere samples at harvest (see Supplementary Table 4, sheet “taxtable,” column “insyncom”). Although strain-level identity cannot be confirmed by 16S rRNA gene-based identification alone, this suggests that taxa that matched the SynCom members continued to exist in the rhizosphere.

### 3.3 Limited treatment effects on barley leaf transcriptomes after batch correction

**FIGURE 3:**
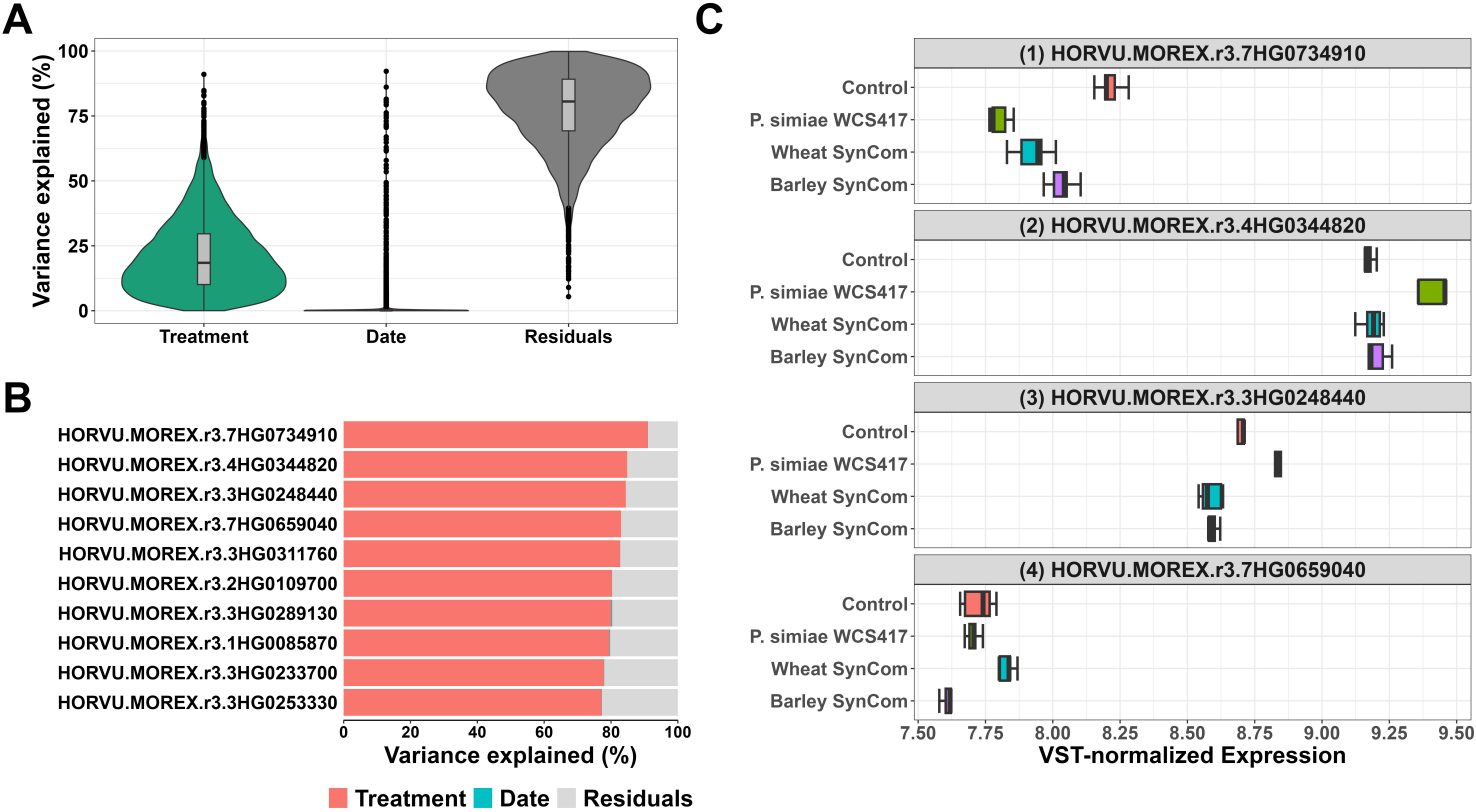
Variance partitioning of gene expression by treatment in barley leaves subjected to four treatments (mock/control, WCS417r, barley SynCom, wheat SynCom). Five biological replicates were analyzed per treatment. **(A)** Violin plot showing variance partitioning in barley leaf transcriptomes. The violin plots show the distribution of variance explained across all genes by (i) treatment and (ii) residuals (unexplained variance). Wider regions indicate a greater density of genes with the corresponding proportion of variance explained. Treatments accounted for a small proportion of variance, while most gene expression variation remained unexplained (residuals). **(B)** Bar plot showing the top ten genes with the highest variance explained by treatment, based on variance partitioning using the model ∼ treatment + date. The *x*-axis shows the percentage of variance explained, the *y*-axis lists the gene IDs. Each bar is divided into the variance explained by treatment, date and residuals. **(C)** VST-normalized expression of the four genes with the highest proportion of variance explained by treatment. Boxplots show expression values (*x*-axis) across the four treatments, with each panel representing one gene.

In order to evaluate the pre-infection responses of barley leaves to inoculation, the barley leaf transcriptome was sequenced to analyze gene expression levels following inoculation with a 10 mM MgCl2 mock control, WCS417r, or SynCom from barley or wheat rhizospheres. PERMANOVA of the batch-corrected profiles revealed no significant differences between the treatments (adjusted *p* > 0.05; *R*^2^ values in Supplementary Table 5 and Supplementary Figure 2). After correcting for batch effect, variance partitioning (Figure 3A) eliminated the effects of sampling date and slightly increased the effects of the treatment (typically around 12% but extending up to 60% for a subset of genes), with residuals dominating variability. The top 10 genes for which differences in gene expression were the most explained by the treatment had variance explained values between 78% and 90% (Figure 3B), with the first four of these genes being annotated as Endo-1,4-beta-xylanase A (HORVU.MOREX.r3.7HG0734910), receptor kinase 1 (HORVU.MOREX.r3.4HG0344820), Ras-like protein (HORVU.MOREX.r3.3HG0248440), and Formin-like protein (HORVU.MOREX.r3.7HG0659040) (Figure 3C).

In summary, treatment did not induce a transcriptome-wide response in barley leaves, however, effects related to the treatment were detectable for a small subset of genes, with annotations linked to cell wall modification, signaling, and cytoskeleton-associated functions.

### 3.4 Bioinoculant treatments only induce subtle transcriptional responses in barley leaves

**FIGURE 4:**
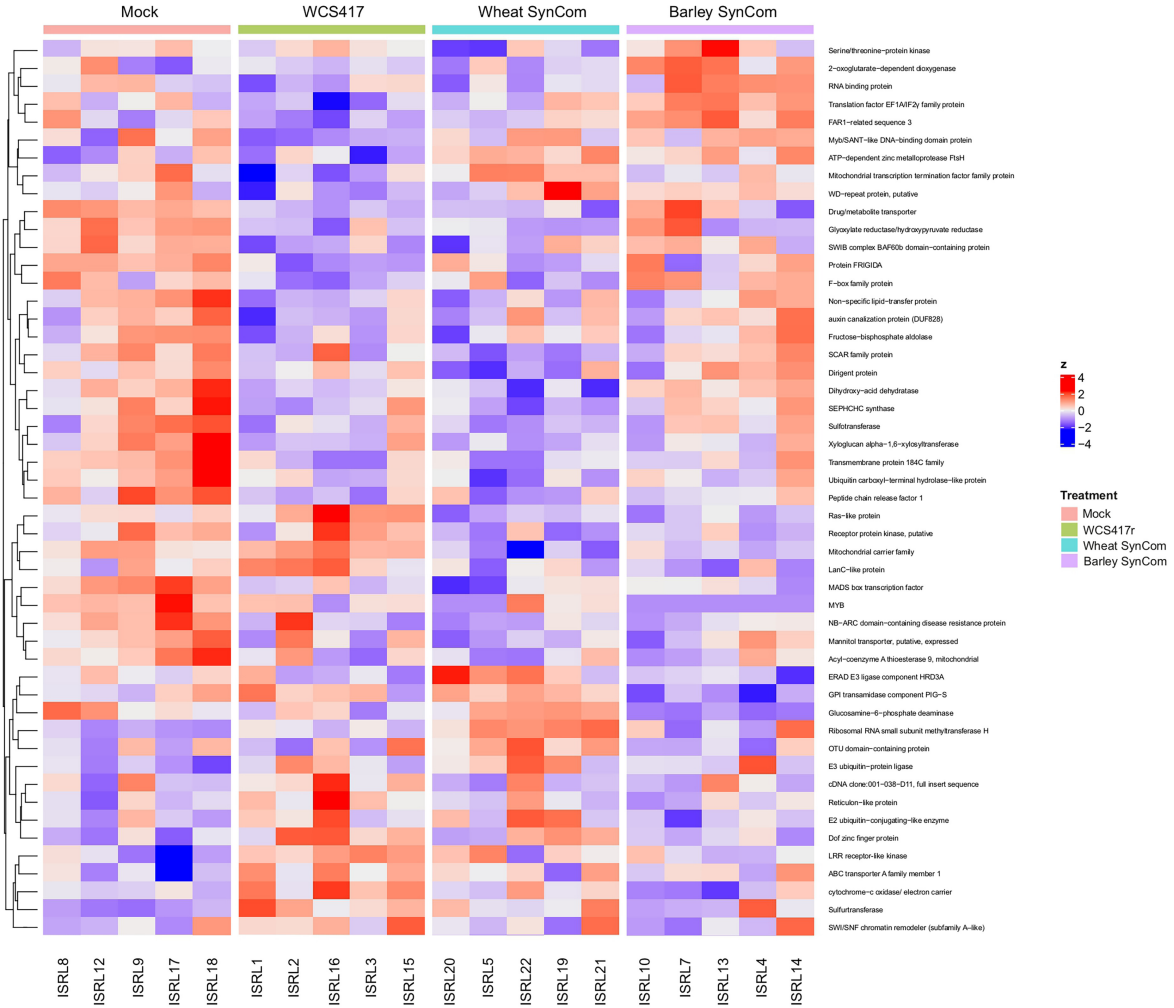
A clustered heatmap showing normalized gene expression levels of 50 out of 200 genes that explain the maximum variance in barley leaf gene expression after inoculation with WCS417r, barley SynCom or wheat SynCom compared to the mock-treated control group. Five biological replicates were analyzed per treatment.

Inoculation with barley SynCom, wheat SynCom, or WCS417r yielded few differentially expressed genes (DEGs) in barley leaves. PERMANOVA confirmed that the treatment had minimal impact on barley gene expression (adjusted *p* > 0.05; Supplementary Table 5), with DESeq2 revealing the fewest DEGs for barley SynCom vs. control (7 genes), then wheat SynCom (54 genes) and WCS417r (110 genes) vs. control (Supplementary Tables 6–8). Variance partitioning further explored these subtle transcriptional responses. Figure 4 summarizes 50 annotated genes selected from the 200 most treatment-responsive genes, selected by treatment-associated expression patterns and annotations (Supplementary Table 9). In barley SynCom-inoculated plants, genes annotated as FAR1-related sequence 3, SCAR family protein, RNA-binding protein, and translation elongation/initiation factor showed higher relative expression, whereas genes annotated as MYB and Dof zinc finger proteins showed lower relative expression. In WCS417r-inoculated plants, genes annotated as mitochondrial carrier family and LanC-like protein showed higher relative expression, whereas a metalloprotease FtsH gene showed lower relative expression. In wheat SynCom-inoculated plants, genes annotated as ERAD E3 ubiquitin ligase HRD3A and E3 ubiquitin-protein ligase showed higher relative expression. A separate GO enrichment analysis for Wheat SynCom versus control is presented in Supplementary Figure 3, highlighting enrichment of terms related to phosphorylation, kinase activity, and protein modification.

### 3.5 Rhizosphere metatranscriptomes show similar taxonomic and functional profiles across treatments

**FIGURE 5:**
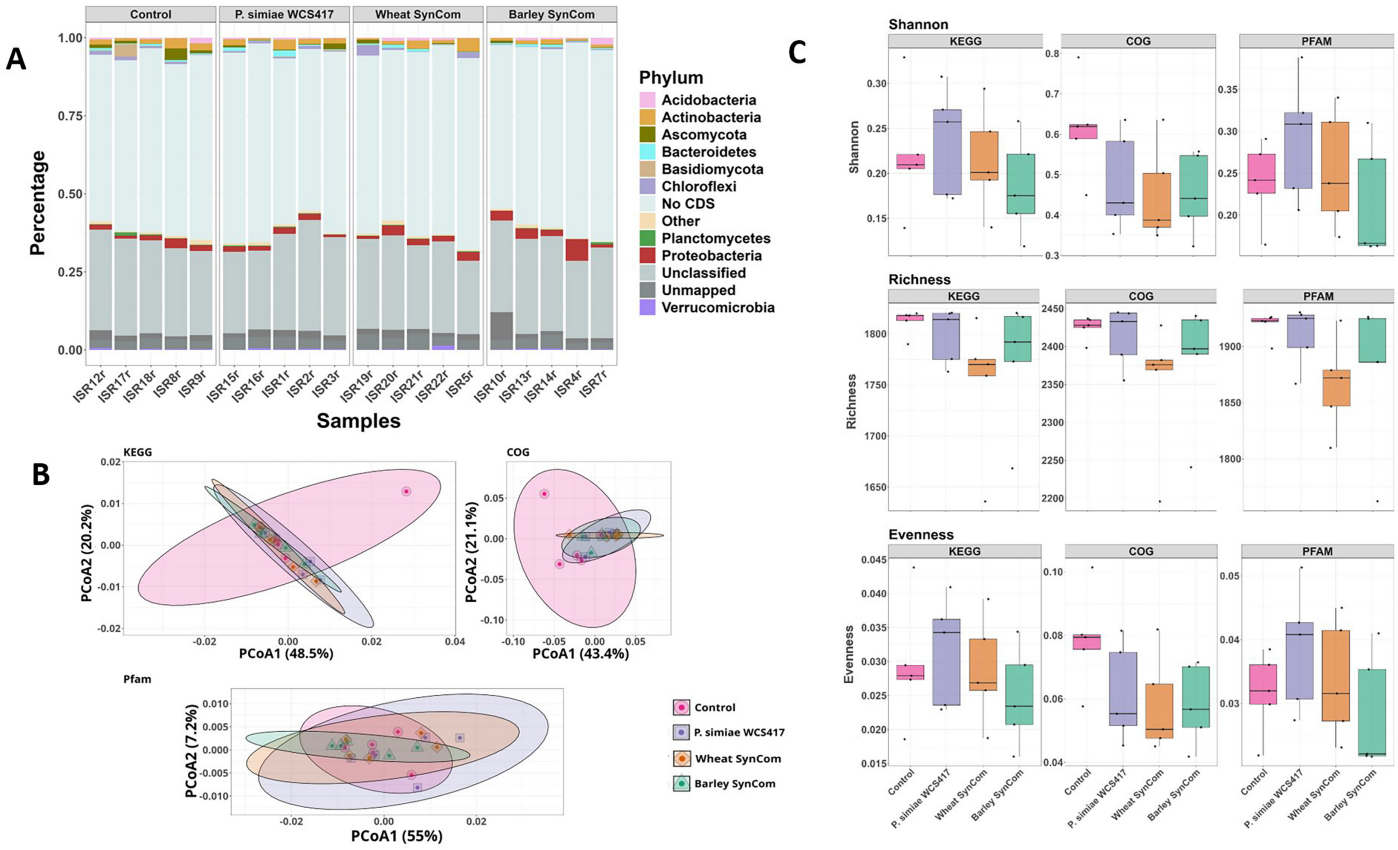
Metatranscriptomic profiles of the barley rhizosphere inoculated with WCS417r, Barley SynCom, and Wheat SynCom in comparison to controls. **(A)** Taxonomic composition across different treatments, showing the relative abundance (%) of 13 phyla, five replicates per treatment. **(B)** Principal coordinate analysis (PCoA) based on KEGG **(A)**, COG **(B)**, and Pfam **(C)** annotations. Axes indicate the percentage of variance explained by PC1 and PC2. **(C)** Alpha-diversity metrics (Shannon, richness, and evenness) for KEGG, COG, and PFAM functional profiles, calculated from relative abundances. Pairwise differences between treatments were assessed using Dunn’s test with false discovery rate (FDR) correction. No significant pairwise differences were detected.

To assess treatment-associated effects on rhizosphere functional activity independently of pathogen challenge, the rhizosphere metatranscriptomes were analyzed from plants that had been treated with a control, WCS417r, or a barley or wheat SynCom. Metatranscriptomic analysis revealed transcripts from 13 phyla across the treatments. Between 30 and 60 percent of reads lacked CDS annotation, followed by “unclassified” and plant host (Streptophyta) mappings. Bacterial phyla showed low, even abundance with no consistent phylum-level differences between treatments (Figure 5A). The metatranscriptomic contigs were assigned based on sequence similarity (not reference SynCom genomes), limiting taxonomy to broad bacterial groups and preventing SynCom strain-level read attribution.

Principal coordinate analysis (PCoA) of KEGG (A), COG (B), and Pfam (C) annotations showed that despite a few outliers among control samples, profiles from all treatments broadly overlapped (Figure 5B). PERMANOVA based on Bray–Curtis distances of relative functional profiles detected no significant treatment effect for KEGG (*R*^2^ = 0.131, *F* = 0.803, *p* = 0.692), COG (*R*^2^ = 0.220, *F* = 1.505, *p* = 0.126), or Pfam (*R*^2^ = 0.147, *F* = 0.916, *p* = 0.514), which is consistent with this pattern. Pairwise PERMANOVA comparisons were likewise not significant after multiple-testing correction (Supplementary Table 10). Alpha diversity was measured with Shannon index, richness, and evenness using KEGG, COG, and Pfam annotations (Figure 5C). The diversity values were similar between all treatments and statistical tests showed no significant differences among pairwise comparisons. A few outliers and variation were present, particularly for Wheat SynCom and Control samples, but no clear pattern related to treatment was detected.

### 3.6 Bioinoculants induce selective functional shifts in rhizosphere metatranscriptomes

**FIGURE 6:**
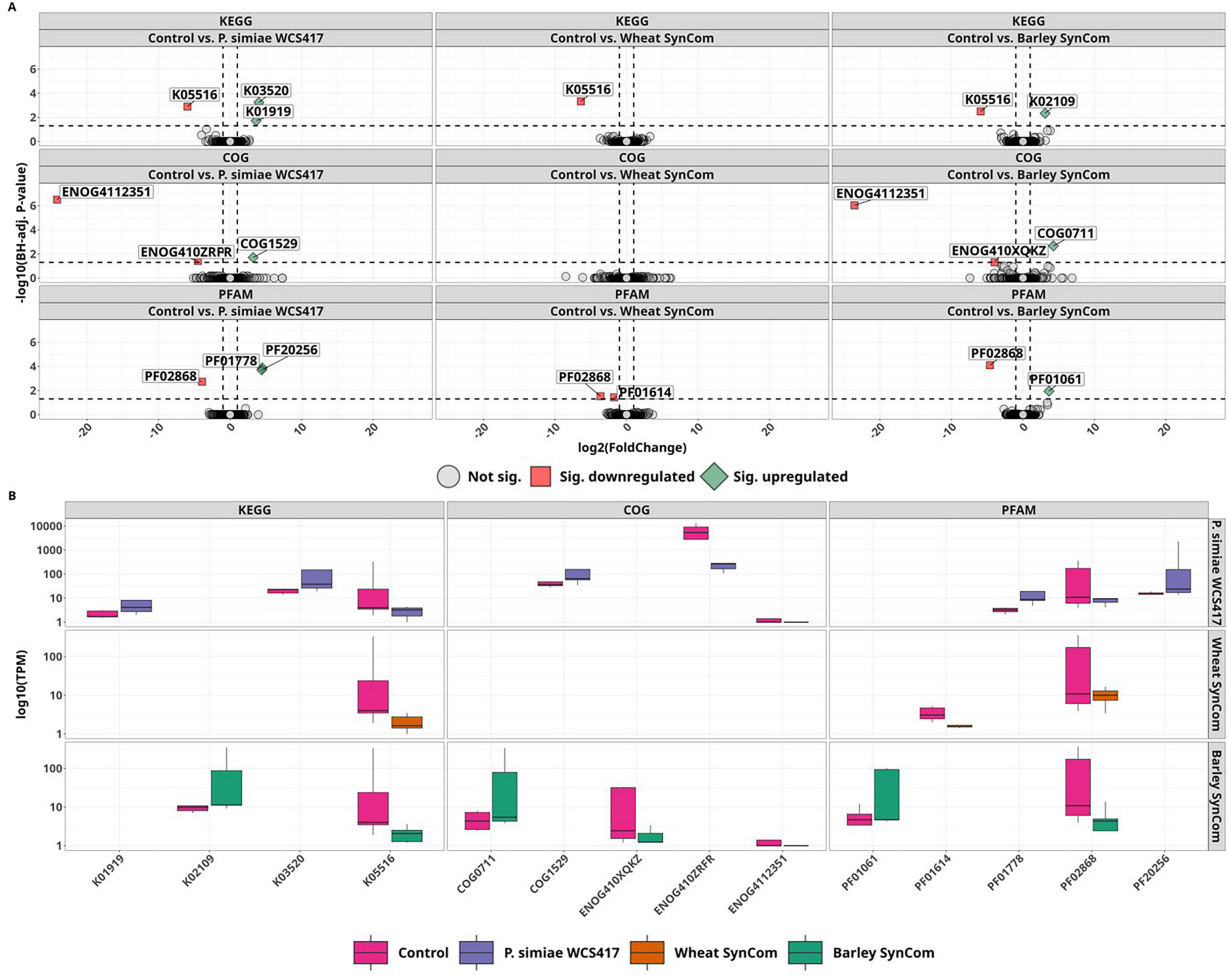
Differential gene expression analysis of barley rhizosphere metatranscriptomes showing significantly upregulated and downregulated functional features. Differentially expressed features were identified using DESeq2 on batch-corrected abundance counts and setting treatment as the explanatory variable. Pairwise contrasts were performed against the control, and significance was defined as Benjamini–Hochberg-adjusted *p*-value (padj) ≤ 0.05 and |log_2_ fold change| ≥ 0.5. **(A)** Volcano plots showing significantly upregulated and downregulated terms across KEGG, COG, and PFAM categories. The *x*-axis represents log_2_(fold change), and the *y*-axis represents –log10(BH-adjusted *p*-value). Significantly changed terms are highlighted compared to all other terms within each category. **(B)** Boxplots of representative significantly upregulated and downregulated terms, displayed by treatment condition. Each boxplot shows log10(TPM) values for the Control versus the respective treatment (e.g., KEGG term K05516 in Control vs. Barley SynCom).

Differential gene expression analysis of the metatranscriptomes revealed 14 functional features (KEGG, COG, and PFAM terms) to be enriched across the bioinoculant applications compared to the controls (BH adjusted *p*-value ≤ 0.05 & absolute log_2_-fold change ≥ 0.5) (Figures 6A,B). WCS417r upregulated genes annotated as K03520 (KEGG), COG1529 (COG), PF01778 (PFAM), and PF20256 (PFAM) that are related to redox-related metabolic functions, ribosomal components, and molybdenum cofactor-binding domains, and also K01919 (glutamate-cysteine ligase). Conversely, WCS417r treatment downregulated ENOG4112351 and ENOG410ZRFR (eggNOG; unknown function), PF02868 (PFAM; thermolysin metallopeptidase–related domain) and K05516 (stress-induced DnaJ-like molecular chaperone).

Inoculation with wheat SynCom resulted in downregulation of PF02868, PF01614 (PFAM; IclR-family transcriptional regulator, C-terminal domain), and K05516 (KEGG; stress-induced DnaJ-like molecular chaperone), while no eggNOG features were significantly downregulated in this treatment. Barley SynCom upregulated K02109 (KEGG; F-type H^+^-transporting ATPase subunit b), COG0711 (COG; ATP synthase–related protein), PF01061 (PFAM; ABC-2 type transporter), and ENOG410XQKZ (eggNOG; oxidoreductase-related domain protein). Several features were downregulated in the barley SynCom treatment, including ENOG4112351 (eggNOG; protein of unknown function) and PF02868 (PFAM; thermolysin metallopeptidase–related domain), and K05516 (KEGG; stress-induced DnaJ-like molecular chaperone). Comparison across treatments revealed that PF02868 and K05516 were significantly downregulated across all treatments, and ENOG4112351 was downregulated under WCS417r and barley SynCom but not wheat SynCom. Overall, bioinoculants induced selective, function-specific shifts in rhizosphere microbial gene expression, not broad community changes.

## 4 Discussion

In this study, we demonstrated that native bacterial SynComs from barley and wheat rhizospheres promote resistance in barley leaves against *Blumeria graminis* f. sp. *hordei* (*Bgh*). Both SynComs reduced *Bgh* propagation in barley and were associated with only subtle leaf transcriptional responses and no statistically significant changes in overall rhizosphere community composition under the tested conditions.

### 4.1 The potential of SynComs as biological control agents against plant pathogens

The effects of SynComs on disease resistance are often context-dependent and are currently less well characterized than those of model ISR strains such as *P. simiae* WCS417r (Pieterse et al., 2021; Pfeilmeier et al., 2024). SynCom-mediated ISR differs from single-strain ISR in that it arises from the interaction of multiple bacterial taxa, which may possess complementary metabolic capabilities, signaling mechanisms and rhizosphere colonization strategies. Our findings therefore extend ISR research beyond the scope of single-strain frameworks to include defined multi-strain communities.

Our work suggests that microbiome-mediated resistance is not strictly dependent on a barley-derived inoculum. DAF staining results showed that all three inoculants reduced *Bgh* growth relative to the mock (*p* < 0.05), with no significant pairwise differences between the inoculants. The lower fluorescence levels observed in our treatments suggest that plants inoculated with beneficial bacteria experienced reduced pathogen growth, which is consistent with previous studies (Lenk et al., 2018, 2019; Brambilla et al., 2022, 2023). Reduced colonization is a common outcome of ISR consistent with ISR-mediated priming in which immune responses are deployed more rapidly upon pathogen attack (Van der Ent et al., 2009; Pieterse et al., 2014; Martinez-Medina et al., 2016). Although our data did not allow direct assignment of ISR to specific JA-, ET-, or SA-dependent pathways, the results demonstrate that defined multi-strain communities can induce disease protection comparable to the model ISR strain WCS417r.

### 4.2 Limited rhizosphere community changes and subtle leaf transcriptional responses after SynCom inoculation

SynCom inoculation did not significantly alter richness or the overall composition of the prokaryote community, though subtle treatment-associated shifts may have remained below detection thresholds. In addition, the microbial composition of the commercial potting substrate was not characterized prior to planting. This represents a limitation of the study since baseline substrate microbiome data would have provided important context for interpreting rhizosphere community assembly and treatment-associated shifts at harvest. The available literature on changes to the rhizosphere microbiome after inoculation with plant beneficial bacteria (PBB) is inconclusive, with some studies reporting increased diversity (Hao et al., 2023) and others reporting no changes (Yim et al., 2025). Despite the fact that inoculation with a bacterial SynCom can induce the expression of numerous genes in roots and shoots (Montoya et al., 2025), we observed minimal transcriptomic differences in barley leaves following SynCom treatments. This is consistent with recent findings on WCS417r-mediated ISR in barley and *A. thaliana* (Sommer et al., 2026; Verhagen et al., 2004), in which phenotypic protection was accompanied by only weak transcriptional changes prior to infection (van de Mortel et al., 2012) and no significant alterations in the expression of genes related to jasmonic acid (JA), ethylene (ET) or salicylic acid (SA) before pathogen challenge (Pieterse et al., 2014; Conrath et al., 2015). It is in contrast, however, with the findings on *Streptomyces* based ISR in oak, where genes related to the three pathways were differentially expressed before the challenge by oak powdery mildew (Kurth et al., 2014). Together, these results support the interpretation that both SynComs induced a primed physiological state in barley without constitutively activating defense-related transcription. Both SynComs and WCS417r were associated with treatment-specific changes in genes linked to transcriptional regulation and cellular response capacity, suggesting partially overlapping but distinct pre-infection transcriptional adjustments in barley leaves. The timing of sampling may also have influenced the strength of the detected responses, as ISR-associated transcriptional changes can be transient and may have been stronger at earlier stages than captured in our pre-infection sampling design (Timmermann et al., 2019).

### 4.3 Rhizosphere microbiome gene expression analysis detects treatment-specific and shared functional changes

In all three treatments, two features were consistently affected: K05516 (stress-induced DnaJ-like molecular chaperone), which was downregulated, and PF02868 (thermolysin metallopeptidase–related domain), which was downregulated, implying stress adaptation and changes in specific functional activities (Yamashino et al., 1994). The wheat SynCom treatment induced fewer transcriptional changes in barley rhizosphere compared to the other treatments, but included the downregulation of PF01614, a bacterial transcriptional regulator, suggesting altered microbial transcriptional regulation (Krell et al., 2006). Inoculation with the barley SynCom was associated with increased expression of ATP synthase-related proteins (K02109, COG0711), ABC-type transporters (PF01061), features linked to stress adaptation and redox-related functions. This implies that there are alterations to microbial energy metabolism, transport and stress-responsive activity in the rhizosphere. At the same time, PF02868 and ENOG4112351 were downregulated, similarly to WCS417r treatment, indicating partly shared functional shifts involving the SynCom and the ISR-eliciting bacterium treatments. In contrast, inoculation with WCS417r was associated with the upregulation of functions related to stress, transport, detoxification, nitrogen metabolism and signaling. This suggests a more responsive and functionally adjusted rhizosphere microbiome rather than a general increase in activity (Pieterse et al., 2021; Andrade et al., 2023; Desrut et al., 2020). Conversely, this treatment was found to downregulate genes related to energy metabolism and ribosomes, suggesting a shift away from growth-related functions and toward more specialized microbial responses (Blazewicz et al., 2013).

The taxonomic overview of the metatranscriptomes revealed that most transcripts could not be assigned to coding sequences and were instead categorized as “unclassified.” This finding is consistent with previous studies and underlines the current limitations of functional and taxonomic assignment in complex rhizosphere datasets (Howe et al., 2023; Jiang et al., 2016). The observed differential expression patterns suggest selective functional responses to bioinoculation instead of widespread shifts across the rhizosphere community. Together these results demonstrate that inoculation with different SynComs and the WCS417r strain induces shifts in the functional activity of rhizosphere microbes, suggesting modulation of energy metabolism, signaling, and nutrient exchange rather than merely growth enhancement.

## 5 Conclusion

Our study demonstrates that SynComs from the rhizosphere of drought-stressed barley and wheat can induce systemic resistance (ISR) in barley against powdery mildew infection. This indicates that ISR can emerge as a community-level property of defined rhizosphere consortia rather than depending solely on individual model ISR strains. The wheat SynCom triggered a response comparable to that induced by the barley SynCom, indicating that communities from related host species can also elicit ISR. Despite containing strains from different genera, all of the bacterial members of the two SynComs belonged to the potential plant growth promoters, including the genera *Pseudomonas*, *Bacillus/Priestia*, *Rhizobium*-related taxa, and *Streptomyces*. The shared PGP traits may in part explain their ability to induce ISR. Meanwhile, treatment-specific differences observed in rhizosphere metatranscriptomes suggest that different microbial communities can achieve similar ISR outcomes via distinct functional and metabolic strategies. The study also detected treatment-specific changes in rhizosphere microbial gene expression, including in response to inoculation with the ISR model strain, *Pseudomonas simiae* WCS417r. These findings provide novel insight into how ISR-eliciting bacteria and communities alter rhizosphere microbial activity beyond effects on host gene expression. Finally, application of SynComs was not associated with changes in host leaf gene expression at the whole-transcriptome level. This is consistent with the priming model of ISR, whereby defense genes remain poised rather than being constitutively active.

## Data Availability

The datasets presented in this study can be found in online repositories. The names of the repository/repositories and accession number(s) can be found below: https://www.ncbi.nlm.nih.gov/, PRJNA1405459.

## Footnotes

1
https://www.ufz.de/index.php?en=42385

## References

[B1] Agrios G. N. (2009). “Plant pathogens and disease: general introduction,” in *Encyclopedia of Microbiology*, 3rd Edn, ed. Schaechter M. (Oxford: Academic Press), 613–646.

[B2] Altermann M. Rinklebe J. Merbach I. Körschens M. Langer U. Hofmann B. (2005). Chernozem—Soil of the Year 2005. *Z. Pflanzenernähr. Bodenk.* 168 725–740. 10.1002/jpln.200521814

[B3] Andrade L. Santos C. H. B. Frezarin E. T. Sales L. R. Rigobelo E. C. (2023). Plant growth-promoting rhizobacteria for sustainable agricultural production. *Microorganisms* 11:1088. 10.3390/microorganisms11041088 37110511 PMC10146397

[B4] Arbizu P. M. (2020). *Pairwiseadonis: Pairwise multilevel comparison using adonis.* Available online at: https://github.com/pmartinezarbizu/pairwiseAdonis (accessed July 14, 2025).

[B5] Barnett D. Arts I. Penders J. (2021). microViz: an R package for microbiome data visualization and statistics. *JOSS* 6:3201. 10.21105/joss.03201

[B6] Bhardwaj N. R. Banyal D. K. Roy A. K. (2021). Prediction model for assessing powdery mildew disease in common Oat (*Avena sativa* L.). *Crop Protect.* 146:105677. 10.1016/j.cropro.2021.105677

[B7] Blazewicz S. J. Barnard R. L. Daly R. A. Firestone M. K. (2013). Evaluating rRNA as an indicator of microbial activity in environmental communities: limitations and uses. *ISME J*. 7 2061–2068. 10.1038/ismej.2013.102 23823491 PMC3806256

[B8] Bolger A. M. Lohse M. Usadel B. (2014). Trimmomatic: a flexible trimmer for Illumina sequence data. *Bioinformatics* 30 2114–2120. 10.1093/bioinformatics/btu170 24695404 PMC4103590

[B9] Brambilla A. Lenk M. Ghirardo A. Eccleston L. Knappe C. Weber B. et al. (2023). Pipecolic acid synthesis is required for systemic acquired resistance and plant-to-plant-induced immunity in barley. *J. Exp. Bot*. 74 3033–3046. 10.1093/jxb/erad095 36905226

[B10] Brambilla A. Sommer A. Ghirardo A. Wenig M. Knappe C. Weber B. et al. (2022). Immunity-associated volatile emissions of β-ionone and nonanal propagate defence responses in neighbouring barley plants. *J. Exp. Bot.* 73 615–630. 10.1093/jxb/erab520 34849759

[B11] Breitkreuz C. Buscot F. Tarkka M. Reitz T. (2020). Shifts between and among populations of wheat rhizosphere *Pseudomonas*, streptomyces and phyllobacterium suggest consistent phosphate mobilization at different wheat growth stages under abiotic stress. *Front. Microbiol*. 10:3109. 10.3389/fmicb.2019.03109 32038552 PMC6987145

[B12] Breitkreuz C. Heintz-Buschart A. Buscot F. Wahdan S. F. M. Tarkka M. Reitz T. (2021a). Can we estimate functionality of soil microbial communities from structure-derived predictions? A reality test in agricultural soils. *Microbiol. Spectr*. 9:e0027821. 10.1128/Spectrum.00278-21 34346741 PMC8552701

[B13] Breitkreuz C. Herzig L. Buscot F. Reitz T. Tarkka M. (2021b). Interactions between soil properties, agricultural management and cultivar type drive structural and functional adaptations of the wheat rhizosphere microbiome to drought. *Environ. Microbiol*. 23 5866–5882. 10.1111/1462-2920.15607 34029439

[B14] Bziuk N. Maccario L. Sørensen S. J. Schikora A. Smalla K. (2022). Barley rhizosphere microbiome transplantation - a strategy to decrease susceptibility of barley grown in soils with low microbial diversity to powdery mildew. *Front. Microbiol*. 13:830905. 10.3389/fmicb.2022.830905 35685930 PMC9173696

[B15] Callahan B. J. McMurdie P. J. Rosen M. J. Han A. W. Johnson A. J. Holmes S. P. (2016). DADA2: high-resolution sample inference from Illumina amplicon data. *Nat. Methods* 13 581–583. 10.1038/nmeth.3869 27214047 PMC4927377

[B16] Caporaso J. G. Lauber C. L. Walters W. A. Berg-Lyons D. Lozupone C. A. Turnbaugh P. J. et al. (2011). Global patterns of 16S rRNA diversity at a depth of millions of sequences per sample. *Proc. Natl. Acad. Sci. U. S. A*. 108 (Suppl. 1), 4516–4522. 10.1073/pnas.1000080107 20534432 PMC3063599

[B17] Chisholm S. T. Coaker G. Day B. Staskawicz B. J. (2006). Host-microbe interactions: shaping the evolution of the plant immune response. *Cell* 124 803–814. 10.1016/j.cell.2006.02.008 16497589

[B18] Cieplak M. Nucia A. Ociepa T. Okoń S. (2022). Virulence structure and genetic diversity of *Blumeria graminis* f. sp. avenae from different regions of Europe. *Plants* 11:1358. 10.3390/plants11101358 35631783 PMC9145444

[B19] Coghlan S. E. Walters D. R. (1992). Photosynthesis in green-islands on powdery mildewinfected barley leaves. *Physiol. Mol. Plant Pathol.* 40 31–38. 10.1016/0885-5765(92)90069-8

[B20] Conrath U. Beckers G. J. Langenbach C. J. Jaskiewicz M. R. (2015). Priming for enhanced defense. *Annu. Rev. Phytopathol*. 53 97–119. 10.1146/annurev-phyto-080614-120132 26070330

[B21] Cowger C. Mehra L. Arellano C. Meyers E. Murphy J. P. (2018). Virulence Differences in *Blumeria graminis* f. sp. tritici from the Central and Eastern United States. *Phytopathology* 108 402–411. 10.1094/PHYTO-06-17-0211-R 29082810

[B22] Czembor J. H. Czembor E. (2023). Sources of resistance to powdery mildew in wild barley (Hordeum vulgare subsp. spontaneum) collected in Jordan, Lebanon, and Libya. *Agronomy* 13:2462. 10.3390/agronomy13102462

[B23] Delgado-Baquerizo M. Guerra C. A. Cano-Díaz C. Egidi E. Wang J.-T. Eisenhauer N. et al. (2020). The proportion of soil-borne pathogens increases with warming at the global scale. *Nat. Clim. Chang.* 10 550–554. 10.1038/s41558-020-0759-3

[B24] Desrut A. Moumen B. Thibault F. Le Hir R. Coutos-Thévenot P. Vriet C. (2020). Beneficial rhizobacteria *Pseudomonas* simiae WCS417 induce major transcriptional changes in plant sugar transport. *J. Exp. Bot*. 71 7301–7315. 10.1093/jxb/eraa396 32860502

[B25] Duan Y. Han M. Grimm M. Schierstaedt J. Imani J. Cardinale M. et al. (2023). Hordeum vulgare differentiates its response to beneficial bacteria. *BMC Plant Biol*. 23:460. 10.1186/s12870-023-04484-5 37789272 PMC10548682

[B26] Ebrahimi-Zarandi M. Saberi Riseh R. Tarkka M. T. (2022). Actinobacteria as effective biocontrol agents against plant pathogens, an overview on their role in eliciting plant defense. *Microorganisms* 10:1739. 10.3390/microorganisms10091739 36144341 PMC9500821

[B27] Elagamey E. Abdellatef M. A. E. Haridy M. S. A. Abd El-aziz E. A. E. (2023). Evaluation of natural products and chemical compounds to improve the control strategy against cucumber powdery mildew. *Eur. J. Plant Pathol.* 165 385–400. 10.1007/s10658-022-02612-9

[B28] Estensmo E. L. F. Maurice S. Morgado L. Martin-Sanchez P. M. Skrede I. Kauserud H. (2021). The influence of intraspecific sequence variation during DNA metabarcoding: a case study of eleven fungal species. *Mol. Ecol. Resour*. 21 1141–1148. 10.1111/1755-0998.13329 33459491

[B29] Fujiwara K. Iida Y. Someya N. Takano M. Ohnishi J. Terami F. et al. (2016). Emergence of antagonism against the pathogenic fungus *Fusarium oxysporum* by interplay among non-antagonistic bacteria in a hydroponics using multiple parallel mineralization. *J. Phytopathol.* 164 853–862. 10.1111/jph.12504

[B30] Glick B. R. (2014). Bacteria with ACC deaminase can promote plant growth and help to feed the world. *Microbiol. Res*. 169 30–39. 10.1016/j.micres.2013.09.009 24095256

[B31] Gu Y. Zavaliev R. Dong X. (2017). Membrane trafficking in plant immunity. *Mol. Plant* 10 1026–1034. 10.1016/j.molp.2017.07.001 28698057 PMC5673114

[B32] Hao X. Wang X. Chen C. Liu R. Yin Y. Yao J. et al. (2023). Synthetic bacterial communities reshape microbial communities and enhance nutrient supply in desertified land of Northwest China. *Appl. Soil Ecol.* 189:104972. 10.1016/j.apsoil.2023.104972

[B33] Hatfield J. L. Prueger J. H. (2015). Temperature extremes: effect on plant growth and development. *Weather Clim. Extremes* 10 4–10. 10.1016/j.wace.2015.08.001

[B34] Heil M. (2001). Induced systemic resistance (ISR) against pathogens – a promising field for ecological research. *Perspect. Plant Ecol. Evol. Systemat.* 4 65–79. 10.1078/1433-8319-00015

[B35] Heil M. Bostock R. M. (2002). Induced systemic resistance (ISR) against pathogens in the context of induced plant defences. *Ann. Bot*. 89 503–512. 10.1093/aob/mcf076 12099523 PMC4233886

[B36] Howe K. L. Seitz K. W. Campbell L. G. Baker B. J. Thrash J. C. Rabalais N. N. et al. (2023). Metagenomics and metatranscriptomics reveal broadly distributed, active, novel methanotrophs in the Gulf of Mexico hypoxic zone and in the marine water column. *FEMS Microbiol. Ecol*. 99:fiac153. 10.1093/femsec/fiac153 36520069 PMC9874027

[B37] Israr A. (2023). Plant growth promotion and nutrient mobilization by indigenous bacteria from soil under long term wheat and maize cultivation in district Bhimber. *Pak. J. Agri. Sci.* 60 83–93.

[B38] Jiang Y. Xiong X. Danska J. Parkinson J. (2016). Metatranscriptomic analysis of diverse microbial communities reveals core metabolic pathways and microbiome-specific functionality. *Microbiome* 4:2. 10.1186/s40168-015-0146-x 26757703 PMC4710996

[B39] Kejela T. (2023). “Phytohormone-producing rhizobacteria and their role in plant growth,” in *New Insights Into Phytohormones*, eds Ali B. Iqbal J. (London: IntechOpen).

[B40] Kim D. Langmead B. Salzberg S. L. (2015). HISAT: a fast spliced aligner with low memory requirements. *Nat. Methods* 12 357–360. 10.1038/nmeth.3317 25751142 PMC4655817

[B41] Kloepper J. W. Ryu C. M. Zhang S. (2004). Induced systemic resistance and promotion of plant growth by Bacillus spp. *Phytopathology* 94 1259–1266. 10.1094/PHYTO.2004.94.11.1259 18944464

[B42] Kloppe T. Boshoff W. Pretorius Z. Lesch D. Akin B. Morgounov A. et al. (2022). Virulence of *Blumeria graminis* f. sp. tritici in Brazil, South Africa, Turkey, Russia, and Australia. *Front. Plant Sci.* 13:954958. 10.3389/fpls.2022.954958

[B43] Krell T. Molina-Henares A. J. Ramos J. L. (2006). The IclR family of transcriptional activators and repressors can be defined by a single profile. *Protein Sci*. 15 1207–1213. 10.1110/ps.051857206 16597823 PMC2242505

[B44] Kurth F. Mailänder S. Bönn M. Feldhahn L. Herrmann S. Große I. et al. (2014). Streptomyces-induced resistance against oak powdery mildew involves host plant responses in defense, photosynthesis, and secondary metabolism pathways. *Mol Plant Microbe Interact*. 27 891–900. 10.1094/MPMI-10-13-0296-R 24779643

[B45] Lane D. J. (1991). *16S/23S rRNA Sequencing. Nucleic Acid Techniques in Bacterial Systematics.* Hoboken, NJ: Wiley.

[B46] Lau J. A. Lennon J. T. (2012). Rapid responses of soil microorganisms improve plant fitness in novel environments. *Proc. Natl. Acad. Sci. U. S. A*. 109 14058–14062. 10.1073/pnas.1202319109 22891306 PMC3435152

[B47] Leach J. E. Triplett L. R. Argueso C. T. Trivedi P. (2017). Communication in the phytobiome. *Cell* 169 587–596. 10.1016/j.cell.2017.04.025 28475891

[B48] Lenk M. Wenig M. Bauer K. Hug F. Knappe C. Lange B. et al. (2019). Pipecolic acid is induced in barley upon infection and triggers immune responses associated with elevated nitric oxide accumulation. *Mol. Plant Microbe Interact*. 32 1303–1313. 10.1094/MPMI-01-19-0013-R 31194615

[B49] Lenk M. Wenig M. Mengel F. Häußler F. Vlot A. C. (2018). *Arabidopsis thaliana* immunity-related compounds modulate disease susceptibility in barley. *Agronomy* 8:142. 10.3390/agronomy8080142

[B50] Liao Y. Smyth G. K. Shi W. (2014). featureCounts: an efficient general purpose program for assigning sequence reads to genomic features. *Bioinformatics* 30 923–930. 10.1093/bioinformatics/btt656 24227677

[B51] Love M. I. Huber W. Anders S. (2014). Moderated estimation of fold change and dispersion for RNA-seq data with DESeq2. *Genome Biol*. 15:550. 10.1186/s13059-014-0550-8 25516281 PMC4302049

[B52] Martinez-Medina A. Flors V. Heil M. Mauch-Mani B. Pieterse C. M. J. Pozo M. J. et al. (2016). Recognizing plant defense priming. *Trends Plant Sci*. 21 818–822. 10.1016/j.tplants.2016.07.009 27507609

[B53] Mbaluto C. M. Zytynska S. E. (2025). Rhizobacteria prime the activation of plant defense and nutritional responses to suppress aphid populations on barley over time. *New Phytol*. 247 2390–2405. 10.1111/nph.70319 40574451 PMC12329176

[B54] McMurdie P. J. Holmes S. (2013). phyloseq: an R package for reproducible interactive analysis and graphics of microbiome census data. *PLoS One* 8:e61217. 10.1371/journal.pone.0061217 23630581 PMC3632530

[B55] Mendes R. Garbeva P. Raaijmakers J. M. (2013). The rhizosphere microbiome: significance of plant beneficial, plant pathogenic, and human pathogenic microorganisms. *FEMS Microbiol. Rev*. 37 634–663. 10.1111/1574-6976.12028 23790204

[B56] Molitor A. Zajic D. Voll L. M. Pons-K Hnemann J. Samans B. Kogel K. H. et al. (2011). Barley leaf transcriptome and metabolite analysis reveals new aspects of compatibility and *Piriformospora indica*-mediated systemic induced resistance to powdery mildew. *Mol. Plant Microbe Interact*. 24 1427–1439. 10.1094/MPMI-06-11-0177 21830949

[B57] Montoya M. Durán-Wendt D. Garrido-Sanz D. Carrera-Ruiz L. Vázquez-Arias D. Redondo-Nieto M. et al. (2025). Functional characterization of a synthetic bacterial community (SynCom) and its impact on gene expression and growth promotion in tomato. *Agronomy* 15:1794. 10.3390/agronomy15081794

[B58] Nievola C. C. Carvalho C. P. Carvalho V. Rodrigues E. (2017). Rapid responses of plants to temperature changes. *Temperature* 4 371–405. 10.1080/23328940.2017.1377812 29435478 PMC5800372

[B59] Niu B. Paulson J. N. Zheng X. Kolter R. (2017). Simplified and representative bacterial community of maize roots. *Proc. Natl. Acad. Sci. U. S. A*. 114 E2450–E2459. 10.1073/pnas.1616148114 28275097 PMC5373366

[B60] Northen T. R. Kleiner M. Torres M. Kovács ÁT. Nicolaisen M. H. Krzyżanowska D. M. et al. (2024). Community standards and future opportunities for synthetic communities in plant-microbiota research. *Nat. Microbiol*. 9 2774–2784. 10.1038/s41564-024-01833-4 39478084

[B61] Oksanen J. Simpson G. L. Blanchet F. G. Kindt R. Legendre P. Minchin P. R. et al. (2025). *vegan: Community Ecology Package.* Finlad: University of Helsinki

[B62] Panke-Buisse K. Poole A. C. Goodrich J. K. Ley R. E. Kao-Kniffin J. (2015). Selection on soil microbiomes reveals reproducible impacts on plant function. *ISME J*. 9 980–989. 10.1038/ismej.2014.196 25350154 PMC4817706

[B63] Paradis E. Claude J. Strimmer K. (2004). APE: analyses of phylogenetics and evolution in R language. *Bioinformatics* 20 289–290. 10.1093/bioinformatics/btg412 14734327

[B64] Pfeilmeier S. Werz A. Ote M. Bortfeld-Miller M. Kirner P. Keppler A. et al. (2024). Leaf microbiome dysbiosis triggered by T2SS-dependent enzyme secretion from opportunistic *Xanthomonas pathogens*. *Nat. Microbiol*. 9 136–149. 10.1038/s41564-023-01555-z 38172620 PMC10769872

[B65] Pieterse C. M. Van der Does D. Zamioudis C. Leon-Reyes A. Van Wees S. C. (2012). Hormonal modulation of plant immunity. *Annu. Rev. Cell Dev. Biol*. 28 489–521. 10.1146/annurev-cellbio-092910-154055 22559264

[B66] Pieterse C. M. van Wees S. C. van Pelt J. A. Knoester M. Laan R. Gerrits H. et al. (1998). A novel signaling pathway controlling induced systemic resistance in Arabidopsis. *Plant Cell* 10 1571–1580. 10.1105/tpc.10.9.1571 9724702 PMC144073

[B67] Pieterse C. M. Zamioudis C. Berendsen R. L. Weller D. M. Van Wees S. C. Bakker P. A. (2014). Induced systemic resistance by beneficial microbes. *Annu. Rev. Phytopathol*. 52 347–375. 10.1146/annurev-phyto-082712-102340 24906124

[B68] Pieterse M. J. Berendsen R. L. Jonge R. Stringlis I. A. van Dijken A. J. H. van Pelt J. A. et al. (2021). *Pseudomonas simiae* WCS417: star track of a model beneficial rhizobacterium. *Plant Soil* 461 245–263. 10.1007/s11104-020-04786-9

[B69] Pikovskaya R. I. (1948). Mobilization of phosphorus in soil connection with the vital activity of some microbial species. *Microbiology* 17 362–370.

[B70] Puente-Sánchez F. García-García N. Tamames J. (2020). SQMtools: automated processing and visual analysis of ‘omics data with R and anvi’o. *BMC Bioinformat*. 21:358. 10.1186/s12859-020-03703-2 32795263 PMC7430844

[B71] Quast C. Pruesse E. Yilmaz P. Gerken J. Schweer T. Yarza P. et al. (2013). The SILVA ribosomal RNA gene database project: improved data processing and web-based tools. *Nucleic Acids Res*. 41 D590–D596. 10.1093/nar/gks1219 23193283 PMC3531112

[B72] Rezaei E. E. Webber H. Asseng S. Boote K. Durand J. L. Ewert F. et al. (2023). Climate change impacts on crop yields. *Nat. Rev. Earth Environ.* 4 831–846. 10.1038/s43017-023-00491-0

[B73] Rigerte L. (2026). *Figure 1. Workflow of the Study, Illustrating the Work Described in the Materials and methods Section 3.1, 3.2 and 3.5 (Created in BioRender).* Created in BioRender. Available online at: https://BioRender.com/9kb2jl6

[B74] Rigerte L. Heintz-Buschart A. Reitz T. Tarkka M. T. (2025). Assembly and application of a synthetic bacterial community for enhancing barley tolerance to drought. *Front. Bacteriol.* 4:1572294. 10.3389/fbrio.2025.1572294

[B75] Sacharow J. Salehi-Mobarakeh E. Ratering S. Imani J. Österreicher Cunha-Dupont A. Schnell S. (2023). Control of *Blumeria graminis* f. sp. hordei on Barley Leaves by treatment with fungi-consuming protist isolates. *Curr. Microbiol*. 80:384. 10.1007/s00284-023-03497-5 37872440 PMC10593611

[B76] Sato H. Mizoi J. Shinozaki K. Yamaguchi-Shinozaki K. (2024). Complex plant responses to drought and heat stress under climate change. *Plant J*. 117 1873–1892. 10.1111/tpj.16612 38168757

[B77] Schädler M. Buscot F. Klotz S. Reitz T. Durka W. Bumberger J. et al. (2019). Investigating the consequences of climate change under different land-use regimes: a novel experimental infrastructure. *Ecosphere* 10:e02635. 10.1002/ecs2.2635

[B78] Schloss P. D. (2021). Amplicon sequence variants artificially split bacterial genomes into separate clusters. *mSphere* 6:e0019121. 10.1128/mSphere.00191-21 34287003 PMC8386465

[B79] Shen G. Zhang J. Lei Y. Xu Y. Wu J. (2023). Between-plant signaling. *Annu. Rev. Plant Biol*. 74 367–386. 10.1146/annurev-arplant-070122-015430 36626804

[B80] Singh B. K. Delgado-Baquerizo M. Egidi E. Guirado E. Leach J. E. Liu H. et al. (2023). Climate change impacts on plant pathogens, food security and paths forward. *Nat. Rev. Microbiol*. 21 640–656. 10.1038/s41579-023-00900-7 37131070 PMC10153038

[B81] Sommer A. Dey S. Knappe C. Wenig M. Heuschmann C. Kublik S. et al. (2026). *Pseudomonas simiae*-induced resistance in barley is associated with compromised JA signaling and phyllosphere microbiome responses. *bioRxiv [Preprint].* 10.64898/2026.01.25.701566 PMC13402475 42516976

[B82] Sommer A. Wenig M. Knappe C. Kublik S. Foesel B. U. Schloter M. et al. (2024). A salicylic acid-associated plant-microbe interaction attracts beneficial Flavobacterium sp. to the *Arabidopsis thaliana* phyllosphere. *Physiol Plant* 176:e14483. 10.1111/ppl.14483 39169536

[B83] Spoel S. H. Dong X. (2012). How do plants achieve immunity? Defence without specialized immune cells. *Nat. Rev. Immunol*. 12 89–100. 10.1038/nri3141 22273771

[B84] Tamames J. Puente-Sánchez F. (2019). SqueezeMeta, a highly portable, fully automatic metagenomic analysis pipeline. *Front Microbiol.* 9:3349. 10.3389/fmicb.2018.03349 30733714 PMC6353838

[B85] Timmermann T. Poupin M. J. Vega A. Urrutia C. Ruz G. A. González B. (2019). Gene networks underlying the early regulation of *Paraburkholderia phytofirmans* PsJN induced systemic resistance in Arabidopsis. *PLoS One* 14:e0221358. 10.1371/journal.pone.0221358 31437216 PMC6705864

[B86] van de Mortel J. E. de Vos R. C. Dekkers E. Pineda A. Guillod L. Bouwmeester K. et al. (2012). Metabolic and transcriptomic changes induced in Arabidopsis by the rhizobacterium *Pseudomonas fluorescens* SS101. *Plant Physiol*. 160 2173–2188. 10.1104/pp.112.207324 23073694 PMC3510139

[B87] Van der Ent S. Van Hulten M. Pozo M. J. Czechowski T. Udvardi M. K. Pieterse C. M. J. et al. (2009). Priming of plant innate immunity by rhizobacteria and beta-aminobutyric acid: differences and similarities in regulation. *New Phytol*. 183 419–431. 10.1111/j.1469-8137.2009.02851.x 19413686

[B88] Van Wees S. C. Van der Ent S. Pieterse C. M. (2008). Plant immune responses triggered by beneficial microbes. *Curr. Opin. Plant Biol*. 11 443–448. 10.1016/j.pbi.2008.05.005 18585955

[B89] Verhagen B. W. Glazebrook J. Zhu T. Chang H. S. van Loon L. C. Pieterse C. M. (2004). The transcriptome of rhizobacteria-induced systemic resistance in arabidopsis. *Mol. Plant Microbe Interact*. 17 895–908. 10.1094/MPMI.2004.17.8.895 15305611

[B90] Verslues P. E. Agarwal M. Katiyar-Agarwal S. Zhu J. Zhu J. K. (2006). Methods and concepts in quantifying resistance to drought, salt and freezing, abiotic stresses that affect plant water status. *Plant J*. 45 523–539. 10.1111/j.1365-313X.2005.02593.x 16441347

[B91] Vlot A. C. Sales J. H. Lenk M. Bauer K. Brambilla A. Sommer A. et al. (2021). Systemic propagation of immunity in plants. *New Phytol*. 229 1234–1250. 10.1111/nph.16953 32978988

[B92] Wang Y. Zhuoma Q. Xu Z. Peng Y. Wang M. (2023). Virulence and genetic types of *Blumeria graminis* f. sp. hordei in tibet and surrounding areas. *J. Fungi* 9:363. 10.3390/jof9030363 36983531 PMC10059672

[B93] Weißbecker C. Schnabel B. Heintz-Buschart A. (2020). Dadasnake, a Snakemake implementation of DADA2 to process amplicon sequencing data for microbial ecology. *Gigascience* 9:giaa135. 10.1093/gigascience/giaa135 33252655 PMC7702218

[B94] Yamashino T. Kakeda M. Ueguchi C. Mizuno T. (1994). An analogue of the DnaJ molecular chaperone whose expression is controlled by sigma s during the stationary phase and phosphate starvation in *Escherichia coli*. *Mol. Microbiol*. 13 475–483. 10.1111/j.1365-2958.1994.tb00442.x 7997164

[B95] Yim B. Heider M. A. Bloem E. Vetterlein D. Behr J. H. Babin D. et al. (2025). Exploring the potential of seed inoculation with microbial consortia to mitigate drought stress in maize plants under greenhouse conditions. *Plant Soil* 1–17. 10.1007/s11104-024-07110-x41523316

[B96] Yu Y. Gui Y. Li Z. Jiang C. Guo J. Niu D. (2022). Induced systemic resistance for improving plant immunity by beneficial microbes. *Plants* 11:386. 10.3390/plants11030386 35161366 PMC8839143

[B97] Zhu L. Huang J. Lu X. Zhou C. (2022). Development of plant systemic resistance by beneficial rhizobacteria: recognition, initiation, elicitation and regulation. *Front. Plant Sci*. 13:952397. 10.3389/fpls.2022.952397 36017257 PMC9396261

