# Supplementary material for: Synthetic rhizosphere bacterial communities induce systemic resistance to barley powdery mildew without major shifts in the native bacterial community
Source: Front Microbiol. 2026 Jun 30;17:1818676. doi: 10.3389/fmicb.2026.1818676 (PMC13365038; doi:10.3389/fmicb.2026.1818676)
Supplement: Supplementary file 11 [file Table_11.docx]

**Supplementary Material**

**Supplementary Figures and Tables**


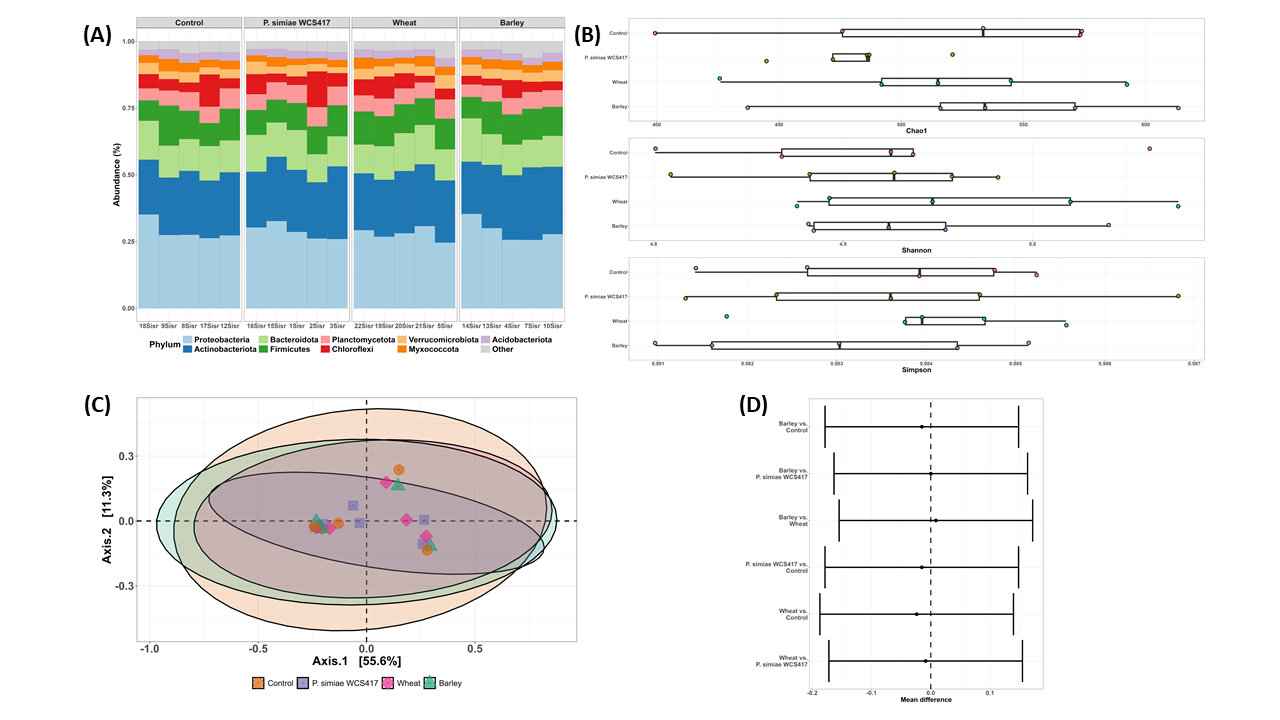


**Supplementary Figure S1. 16S rDNA amplicon sequencing results from soils of barley hosts** treated with a control treatment and three different bioinoculants (*Pseudomonas simiae* WCS417, wheat SynCom, barley SynCom) respectively. (A) Relative abundances of top 10 phyla. (B) Chao1, Shannon, and Simpson indices of alpha diversity. Statistically significant differences between treatments are marked with asterisks. (C) PCoA ordination plot of the samples colored by treatment along the first two principal coordinates. (D) Tukey’s HSD plot for all pairwise comparisons of beta dispersions in the treatments. Error bars that do not include zero (vertical dotted line) and colored red indicate statistically significant differences.


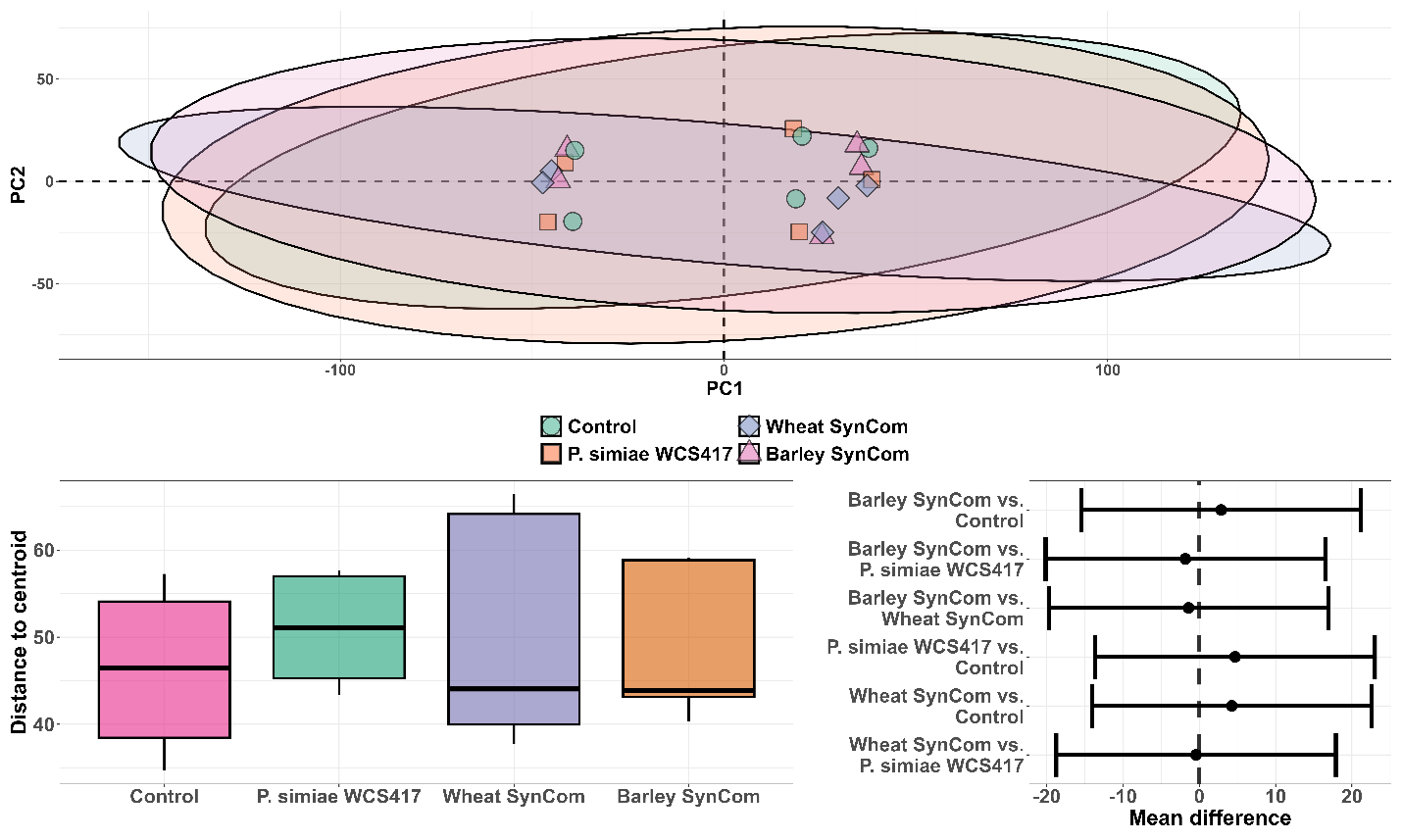


**Supplementary Figure S2. Multivariate analysis of barley leaf transcriptomes** with different inoculations (Control, WCS417, Barley SynCom, Wheat SynCom), barley leaf transcriptomes show no clear treatment separation. (A) PCoA ordination plot of the samples colored by treatment along the first two principal coordinates. (B) Boxplots of the distance to centroid for each treatment, indicating no significant differences. (C) Pairwise PERMANOVA comparisons of treatments, showing mean differences close to zero.

**
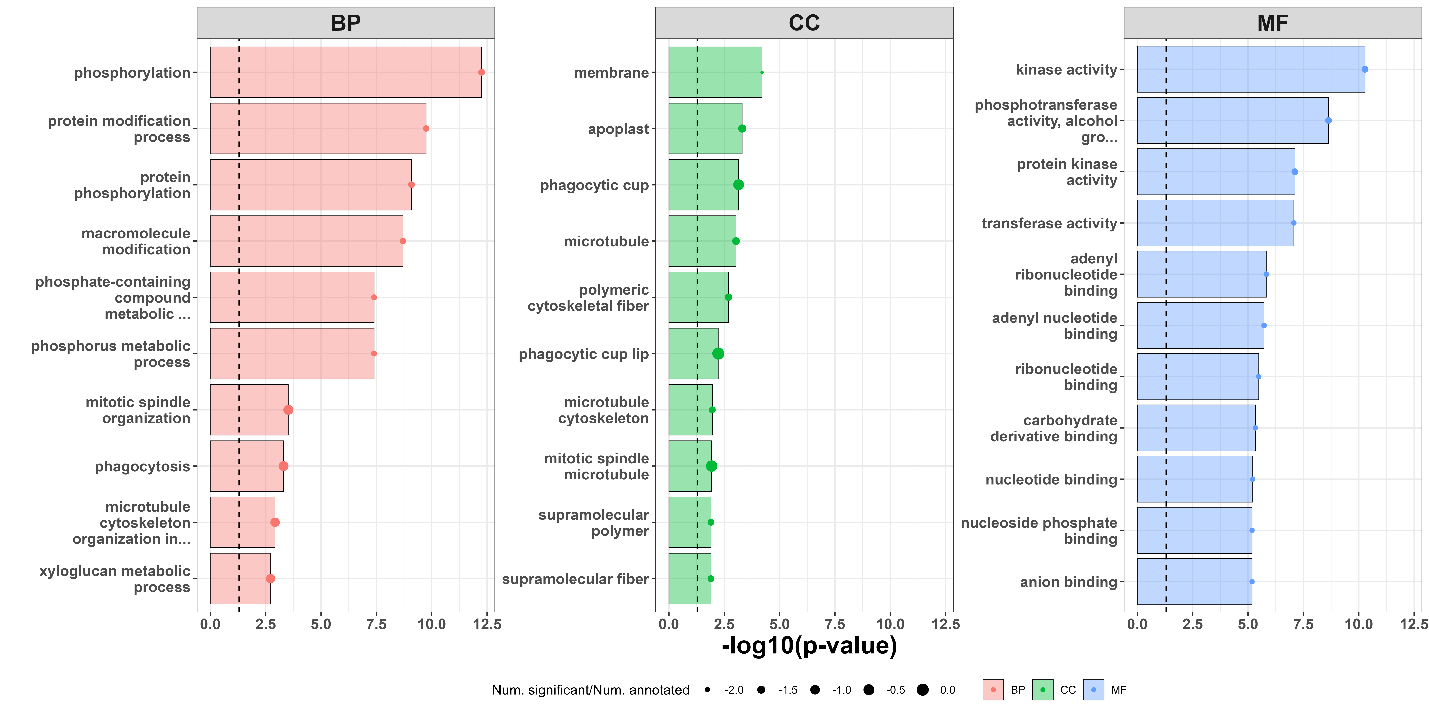
**

**Supplementary Figure S3**. **Gene Ontology (GO) enrichment analysis of barley leaf transcriptomes** inoculated with the Wheat SynCom compared to control plants. Enriched GO terms are shown for the three main categories: Biological Process, Cellular Component, and Molecular Function. The y-axis represents the GO terms, and the x-axis indicates statistical significance as –log10(p-value).
